# Supplementary material for: Non-invasive current collectors for improved current-density distribution during CO2 electrolysis on super-hydrophobic electrodes
Source: Nat Commun. 2023 Oct 18;14:6579. doi: 10.1038/s41467-023-42348-6 (PMC10584973; doi:10.1038/s41467-023-42348-6)
Supplement: Supplementary file 1 — Supplementary Information [file 41467_2023_42348_MOESM1_ESM.docx]

Supplementary Materials for

Non-invasive current collectors for improved current-density distribution during CO_2_ electrolysis on super-hydrophobic electrodes

Hugo-Pieter Iglesias van Montfort^1^, Mengran Li^1,2^, Erdem Irtem^1^, Maryam Abdinejad^1^, Yuming Wu^3^, Santosh K. Pal^1^, Mark Sassenburg^1^, Davide Ripepi^1^, Siddhartha Subramaniam^1^, Jasper Biemolt^1^, Thomas E. Rufford^3^, Thomas Burdyny^1^*

^1^ Department of Chemical Engineering, Delft University of Technology; 9 van der Maasweg, Delft, 2629HZ the Netherlands

^2^ Department of Chemical Engineering, The University of Melbourne; Parkville, Victoria, 3010, Australia

^3^ School of Chemical Engineering, The University of Queensland; St. Lucia 4072, Australia

*Corresponding author. Email: [t.e.burdyny@tudelft.nl](mailto:t.e.burdyny@tudelft.nl)

**This PDF file includes:**

Supplementary Notes

Supplementary Figures 1 to 24

Supplementary Tables 1 to 6

Supplementary References 1 to 11

Supplementary Notes

Reaction-diffusion in a Finite Volume Partial-Differential Equation Solver

In wanting to calculate and represent the effect of the GDE architecture on local availability of CO2 in the catalyst layer, we constructed a 1-D reaction-diffusion model close to the gas-liquid interface of both ePTFE and carbon-based electrodes. To define and execute the solver, we used the FiPy solver package, in a Python environment.^1^

This model is focused on the catalytic interface in a porous GDE and is defined by three domains: (i) the micro-porous layer (MPL), (ii) the catalyst layer and (iii) the electrolyte, simulating the behavior of CO_2_ in an alkaline flow-electrolyzer. The domain of the model is 500 µm thick, the nodal distance of the mesh is 1 nm. For these regions, (i) can be modelled as a flooded (i.e. liquid-phase) domain or a gaseous one, whereas (ii) and (iii) are considered to be wetted throughout. The MPL is assumed to be 20 µm thick for the ePTFE simulation and 40 µm in the case of a carbon GDL.^2,3^ The catalyst layer is 500 nm thick in both cases and the rest of the modelled domain is liquid-phase electrolyte as used in the experiments (1M KOH).

In the gaseous phase (i), gas-species (CO2, CO and H2) are governed by simple diffusion equations, in the form of:

$$\begin{aligned} \frac{\partial c_{i}}{\partial t}=D_{i}\frac{\partial^{2}c_{i}}{\partial t^{2}} \end{aligned}S1$$

With a convective transport boundary on the left limit of the system and a phase-change (gas to liquid) on the right limit. In the catalyst layer, dissolved species (CO_2,aq_, OH^-^, HCO_3_^-^, CO_3_^2-^ and H^+^) are governed by:

$$\begin{aligned} \frac{\partial c_{i}}{\partial t}=D_{i}\frac{\partial^{2}c_{i}}{\partial t^{2}}+R_{h}+R_{i} \#S2 \end{aligned}$$

where R_h_ are the homogeneous CO_2_-buffering reactions:

$$\begin{aligned} CO_{2}+OH^{-}\rightleftharpoons HCO_{3}^{-}\#S3 \end{aligned}$$

$$\begin{aligned} HCO_{3}^{-}+OH^{-}\rightleftharpoons CO_{3}^{2-}\#S4 \end{aligned}$$

and R_i_ are the electrochemical production and consumption rates of CO_2_, OH^-^, CO and H_2_:

$$\begin{aligned} R_{CO_{2}}=-\frac{j_{t}}{F}\left( \frac{s_{CO}}{z_{CO}} \right)\frac{\varepsilon_{cat}}{L_{cat}} \#S5 \end{aligned}$$

$$\begin{aligned} R_{CO}=\frac{j_{t}}{F}\left( \frac{s_{CO}}{z_{CO}} \right)\frac{\varepsilon_{cat}}{L_{cat}} \#S6 \end{aligned}$$

$$\begin{aligned} R_{OH}=\frac{j_{t}}{F}\frac{\varepsilon_{cat}}{L_{cat}} \#S7 \end{aligned}$$

$$\begin{aligned} R_{H_{2}}=\frac{j_{t}}{F}\left( \frac{{1-s}_{CO}}{z_{H_{2}}} \right)\frac{\varepsilon_{cat}}{L_{cat}} \#S8 \end{aligned}$$

where s_i_ are the selectivities (faradaic efficiencies) towards every product (assumed to be 80% CO and 20% H_2_), ε_cat_ is the porosity of the catalyst layer, L_cat_ its length, F the Faraday constant, z_i_ the stoichiometric number of electrons involved in each reduction reaction, and j_t_ the total applied current (a scalar in the model). In the liquid phase of the model, iii, only the homogeneous buffering reactions are involved.

A cornerstone of the modelled quantities lies in the amount of aqueous CO_2_ at the gas-liquid interface. Solubility of CO_2_ is heavily influenced by the presence of ions in the liquid phase.^4^ In order to set the calculated [CO_2,aq_] at this interface, the Hessian of the model is disturbed at the nodes before and after the phase-change using a mask involving a large number (2^63^). The CO_2,aq_ concentration is set using the Séchenov constants on the classical Henry model of carbon dioxide dissolution.^5,6^ First, the Henry equation for CO_2_ as a function of temperature:^7^

$$\begin{aligned} \left[ CO_{2,aq} \right]={P_{CO_{2}}\cdot K}_{H}{\cdot K}_{c}\#S9 \end{aligned}$$

With:

$$\begin{aligned} \ln K_{H}=93.4517\left( \frac{100}{T} \right)-60.2409+23.3585\ln\left( \frac{T}{100} \right)\#S10 \end{aligned}$$

The Henry equilibrium is corrected for the presence of ions in the solution, following:^8^

$$\begin{aligned} \log\left( \frac{\left[ CO_{2,aq,0} \right]}{\left[ CO_{2,aq} \right]} \right)=K_{c}C_{i} \#S11 \end{aligned}$$

The correction factor for each ion, K_c_ is calculated using:

$$\begin{aligned} K_{c}= \sum\left( h_{i}+h_{g} \right) \#S12 \end{aligned}$$

$$\begin{aligned} h_{g}=h_{g,0}+h_{T}\left( T-298.15 \right)\#S13 \end{aligned}$$

The constants used in this case for each ionic species are taken from earlier reports in literature. The concentration of K^+^ is calculated imposing electronic neutrality at the boundary each solving loop, bicarbonate anions are disregarded since their concentration is residual.

**Supplementary Table 1.** Séchenov constants for involved ionic species.

| **Species** | **h_i_** |
| --- | --- |
| K^+^ | 0.0922 |
| OH^-^ | 0.0839 |
| CO_3_^2-^ | 0.1423 |

**Supplementary Table 2.** Correction factors for CO_2_ dilution and temperature effects.

| **Magnitude** | **Value** |
| --- | --- |
| h_g,0_ | -0.0172 |
| h_T_ | -0.000338 |

The value of [CO_2,aq_] is updated every computational loop of the model assuming a partial pressure of 1.1 bar for CO, and is the basis of the boundary value of the plots in Fig 1d and e.

Working principle of IR thermography on gas-diffusion electrodes for CO_2_RR

While we have detailed the working principles of infrared thermography for electrochemical activity mapping in previous works,^9^ the text below reproduces the rationale behind it in order to illustrate the design considerations for this case.

For infrared thermography to be a valid technique for this use case, we lay 3 main conditions that must be met in order to couple temperature measurements to local electrochemical activity. These are:

1. *Heat production must be sufficient so that activity can be mapped at relevant current densities*

Power consumed by an electrochemical system is defined as:

$$\begin{aligned} P=I\cdot\Delta E\#S14 \end{aligned}$$

Applying Ohm’s law, this translates to:

$$\begin{aligned} P=I^{2}\cdot R \#S15 \end{aligned}$$

At an electrode, a part of this power will be destined to driving the reaction, whereas parts of it will be translated into heating of the system’s surrounding. This heating, in electrolysis, is mainly due to either ohmic resistances or reaction overpotential. For the latter, the heat stream is proportional to the reaction overpotential, which in its turn is the difference between the required potential (E_app_) and the thermoneutral potential of the reaction in question (E_tn,0_), by:

$$\begin{aligned} Q_{cat}=\left( E_{app}-E_{tn.0} \right)\cdot I \#S16 \end{aligned}$$

$$\begin{aligned} q_{cat}^{''}=\frac{Q_{cat}}{A_{geo}}=\left( E_{app}-E_{tn.0} \right)\cdot j \#S17 \end{aligned}$$

If we take, for example, reduction of CO_2_ to CO (simplified process of the reactions at a copper electrode, with E_tn,0_ = 0.264 V vs. RHE) and compare it to the operational, i·R corrected potential of the tested ePTFE electrodes at 50 mA cm^-2^ (~ –0.65 V vs. RHE), the total heat generation will be:

$$\begin{aligned} q_{cat,Cu}^{''}=\left( E_{app}-E_{tn.0} \right)\cdot j=\left( -0.65-0.264 \right)\cdot\left( -0.05 \right)= 0.046 W cm^{-2}\#S18 \end{aligned}$$

At the same current density, the contribution to heating of the other main source, ohmic resistance, is calculated by considering the measured resistance between the GDE and the RE (in this case, 0.48 Ω):

$$\begin{aligned} q_{ohm}^{''}=I^{2}R=\left( j\cdot A \right)^{2}\cdot R=\left( -0.05\cdot5 \right)^{2}\cdot0.48=0.030 W cm^{2}\#S19 \end{aligned}$$

which is lower than the heating resulting from the reaction overpotential, thus fulfilling requirement *i*.

1. *Heat generation must be translatable to temperature*

The heat produced, will not translate itself to observational temperature increases if the specific heat of the GDE’s backbone is too high. The C_p,m_ value for ePTFE is around 1500 J kg^-1^K^-1^.^10^Assuming an area weight for our electrodes similar to that of a thick sheet of paper (120 g m^-2^, or 1.2·10^-5^ kg cm^-2^), we can calculate a theoretical heating rate by:

$$\begin{aligned} \frac{\Delta T}{t}=\frac{Q_{cat}}{m\cdot C_{p,m}}=\frac{q_{cat}^{''}}{m^{''}\cdot C_{p,m}}=\frac{0.046 W cm^{-2}}{\left( 1.2\cdot10^{-5} kg cm^{-2} \right)\cdot(1500 J kg^{-1} K^{-1})}=2.55 K s^{-1} \#S20 \end{aligned}$$

Which constitutes a heating rate even higher to the one we previously reported.^9^ This second requirement is thus also met.

1. *The temperature of the electrode’s backbone is representative of the catalyst’s temperature*

The final topic to assess the viability of this technique is the representativity of the backbone’s temperature with respect to the catalyst’s temperature. In case not enough heat is transported through the GDE to its open back side and is otherwise advectively removed by the flowing electrolyte, the measured temperature is not representative of the activity at a certain location. To compare the two heat streams (towards the backbone of the GDE and towards the electrolyte layer), we can devise a simplified 1-D heat conduction problem based on Fourier’s law of heat conduction:

In evaluating the ratio between the heat fluxes, we can quantify how much heat ‘travels’ to the back of the GDE, that is filmed by the camera, and how much is lost to the advective effect of the electrolyte. For each heat flux, we approximate the value by:

$$\begin{aligned} q^{'}=k\nabla T \approx k\frac{\Delta T}{\Delta x}\#S21 \end{aligned}$$

Applying this approximation to the problem described in the sketch above, we obtain:

$$\begin{aligned} Q_{gdl}= \frac{\Delta T}{R_{gde}}=k_{gde}\frac{\Delta T}{\Delta x}A=k_{gde}\frac{(T_{cat}-T_{back,gde})}{\delta_{gde}}A\#S22 \end{aligned}$$

$$\begin{aligned} Q_{el}= \frac{\Delta T}{R_{el}}=k_{el}\frac{\Delta T}{\Delta x}A =k_{el}\frac{(T_{cat}-T_{el})}{\delta_{el}}A\#S23 \end{aligned}$$

$$\begin{aligned} \frac{Q_{gdl}}{Q_{el}}=\frac{{q'}_{gde}}{{q'}_{el}}=\left| \frac{k_{gde}}{k_{el}}\frac{\left( T_{cat}-T_{back,gde} \right)}{\left( T_{cat}-T_{el} \right)}\frac{\delta_{el}}{\delta_{gde}} \right| \#S24 \end{aligned}$$

Where δ is the thickness of each phase, T_cat_ the temperature of the catalyst layer, and k the heat-transfer constant of each phase.

For the solid, GDE-phase, the thermal conductivity can be taken as that of ePTFE, which is around 0.29 W m^-1^ K^-1^.^10^ The thickness of the GDE is around 220 µm total. For the electrolyte, on the other hand, thermal conductivity is assumed, for simplicity, to be equal to that of water (~ 0.6 W m^-1^ K^-1^), while the thermal diffusivity length requires further evaluation. If we assume the Prandtl number of the electrolyte to be close to that of water (~ 7.5), we know that:^11^

$$\begin{aligned} \delta_{elec}=5.0\sqrt{\frac{\nu\cdot x}{u_{0}}}{Pr}^{-\frac{1}{3}} \#S25 \end{aligned}$$

where *v* represents the kinematic viscosity of water, x is the distance that the flow has travelled along the flat plate, and u_0_ is the flow at the channel center.

For the system we discuss, typical flow rates of catholyte used where in the order of 5 sccm. For an electrolyte chamber of dimensions 22 x 22 x 3 mm, this results in an average fluid velocity (u_0_) of 0.13 cm s^-1^. After an average travel distance of 1.1 cm in the catholyte chamber, the thermal boundary between the GDE and the flowing catholyte is around 7400 µm. Filling then out equation S23, we obtain that, in equilibrium (∆T = 1), the ratio of heat fluxes is 16:1 in the direction of the GDE backbone, which confirms that this value of temperature is relevant and representative for the temperature of the catalyst layer.

For further and more intensive analysis of the rationale behind IR thermography for electrochemical activity mapping in electrolysis devices, we kindly refer the reader to our previous work, which includes an intensive analysis in its supporting information.^9^

The rationale for a non-invasive current collector (NICC)

Scaling an electrode design to industrially relevant dimensions poses a challenge, as evidenced by the activity distribution measurements reported in this work. The high ohmic resistance posed by a thin catalyst layer only becomes more dramatic considering the high amounts of current this layer would have to conduct for big electrode sizes. This section outlines the rationale of using a non-invasive current collector (NICC) for scaling purposes.

Let's assume we compare a 2 cm x 2 cm electrode with area 4 cm^2^ (whose busbars are the same as our experimental 2.25 cm x 2.25 cm electrodes) with a 100-fold larger electrode. This would be a 20 cm x 20 cm electrode with an area of 400 cm^2^ (see below Fig. S21). By maintaining the same scaling factors as in our prototype reported in this manuscript and using a 2 cm x 2 cm electrode as the base case, we can expect the 100-fold larger electrode to have a busbar spacing of 20 mm, a busbar height of 100 µm, and a busbar width of 3 mm. The scaling principle we propose here is to scale the busbar dimensions based on the area increase of 100-fold, not the side length dimension increase of 10-fold. The approximate cross-sectional area of the busbars is then 0.3 mm2. The current that travels through each busbar from the outside to the centre would then I = (10 cm) x (2 cm) x (0.2 A cm^-2^) = 4 A (for j = 0.2 A cm^-2^). Segmenting the 10 cm pathway into 20 parts (because current decreases along the length of the busbar), we can calculate the experienced voltage drop by the current to get to a certain point in the electrode using:

$$\begin{aligned} \Delta U_{i}\mathcal{=l\cdot}I_{i}\cdot\frac{\rho}{A} \#S26 \end{aligned}$$

where *l* is the distance from the current collector, *I* the current passing through the segment, *ρ* the approximate resistivity of the copper busbar (1.724x10^‑6^ Ω cm, see Supplementary Table 3) and *A* the cross-sectional area of a busbar. The total voltage drop is then:

$$\begin{aligned} \Delta U_{T}=\sum\Delta U_{i} \#S27 \end{aligned}$$

As shown in Supplementary Figure 21, the voltage drop from the exterior current collector to the center of the small and large electrodes can then be maintained as ~80 mV with a reasonable busbar scaling. Reducing busbar spacing and cross-hatching are added ways to reduce voltage drop and/or the required busbar diameters further.


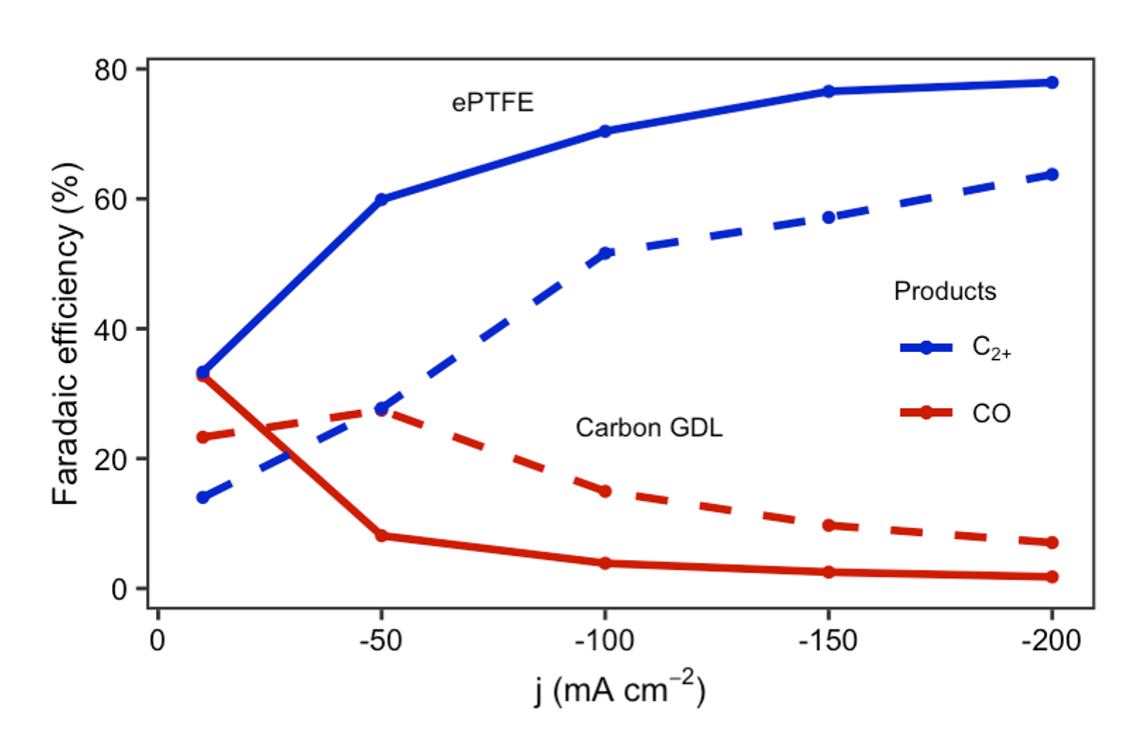


Supplementary Figure 1

Product distribution at increasing current densities for a 200 nm Cu catalyst layers on a carbon GDL (Sigracet® 38BB) and a 500 nm layer on an ePTFE electrode (Sterlitech® Aspire QL822).


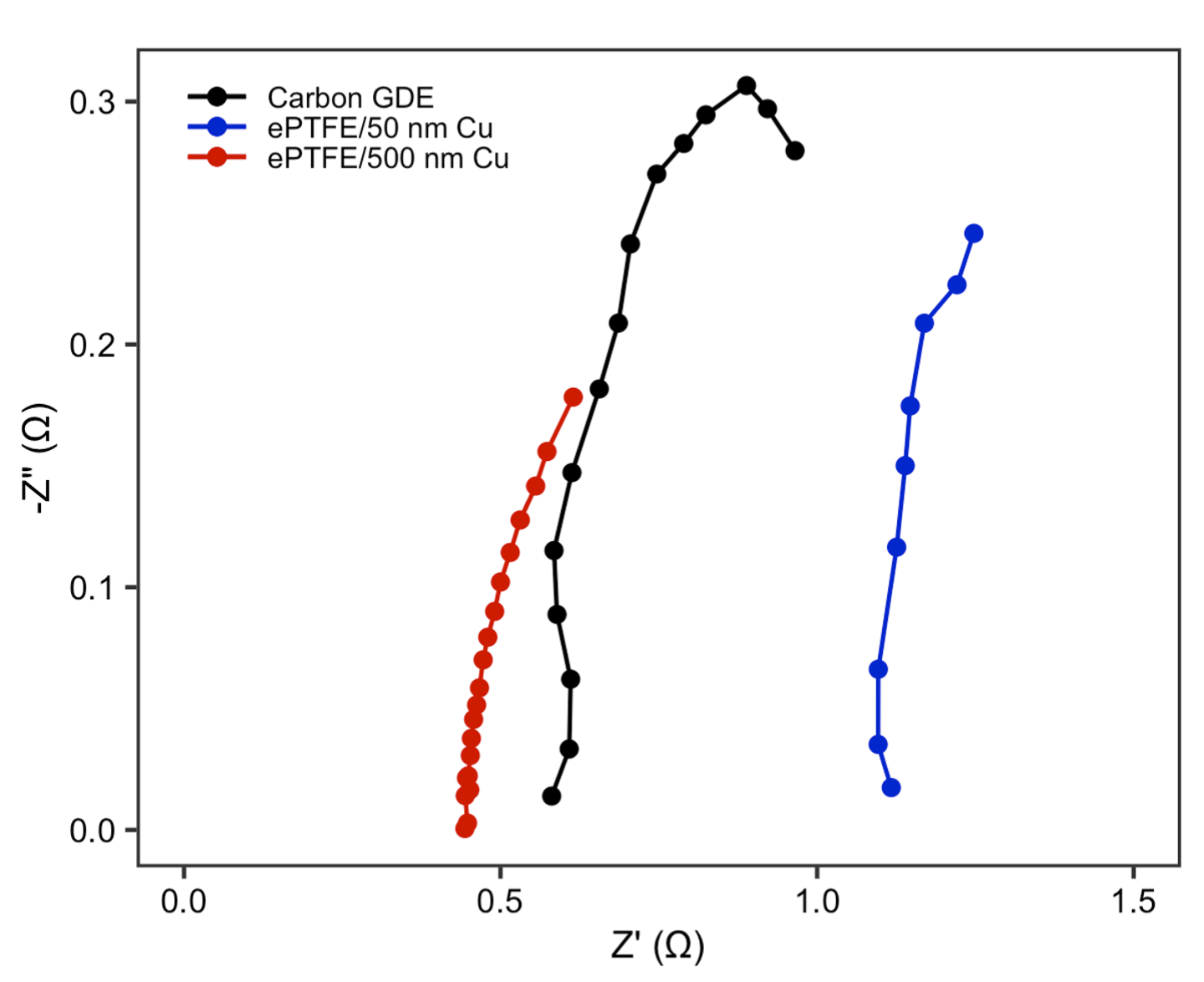


Supplementary Figure 2

High-frequency segments of EIS measurements performed on the three compared electrodes, at a current density of –50 mA cm^-2^ and 1M KOH as the electrolyte.


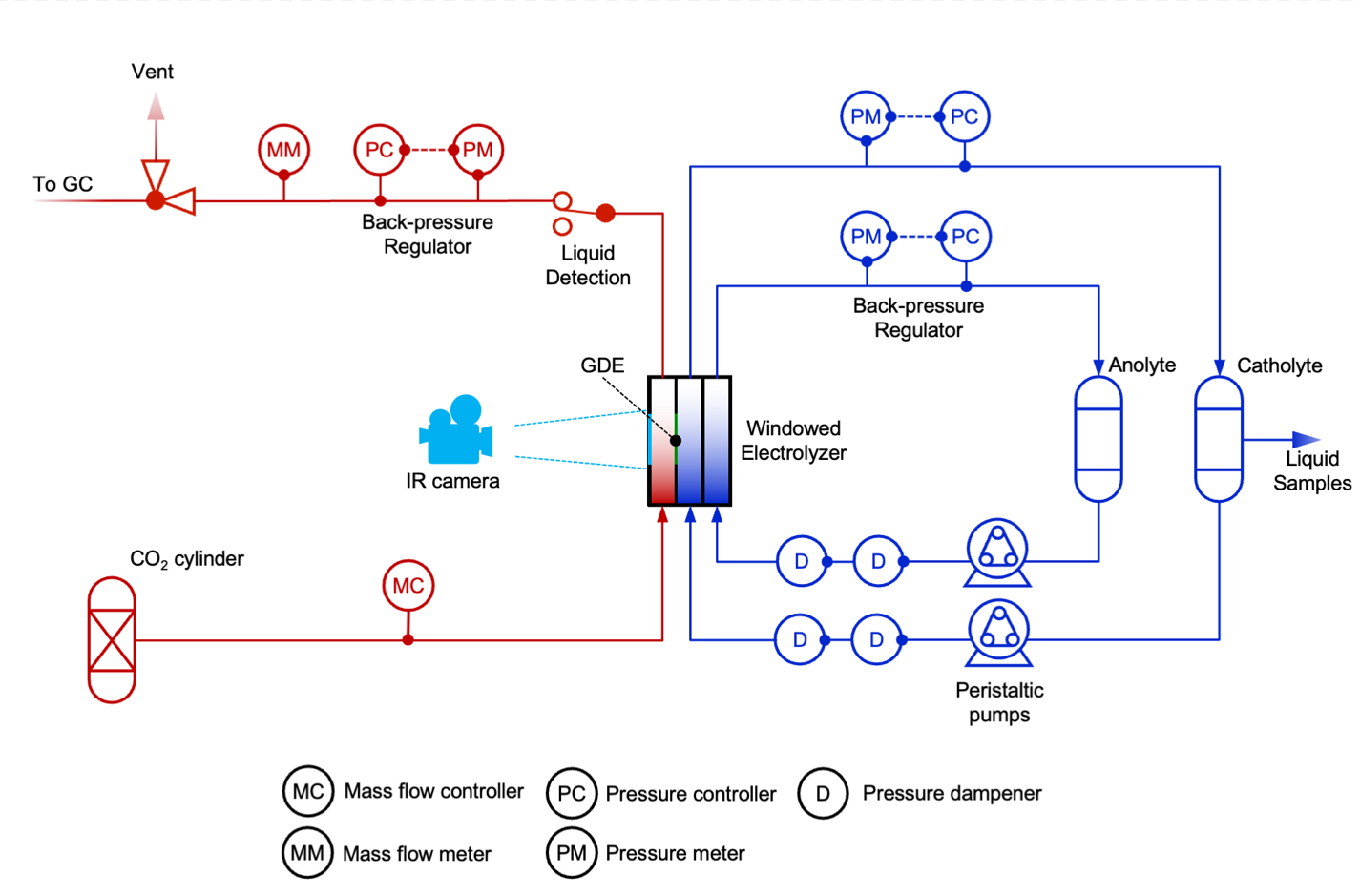


Supplementary Figure 3

Process flow-diagram of the electrochemical testing setup used.


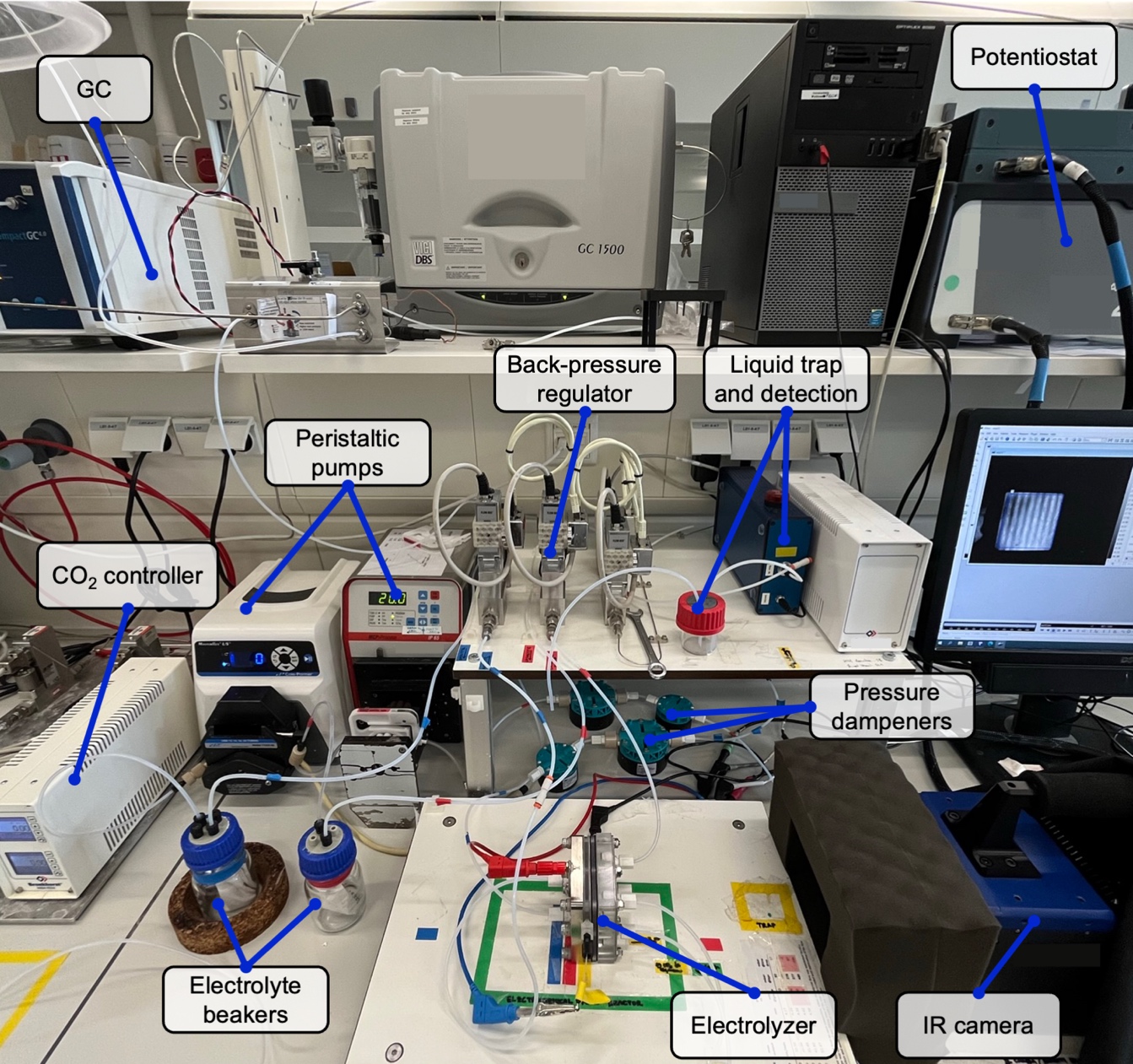


Supplementary Figure 4

Picture of the electrochemical testing bench used.


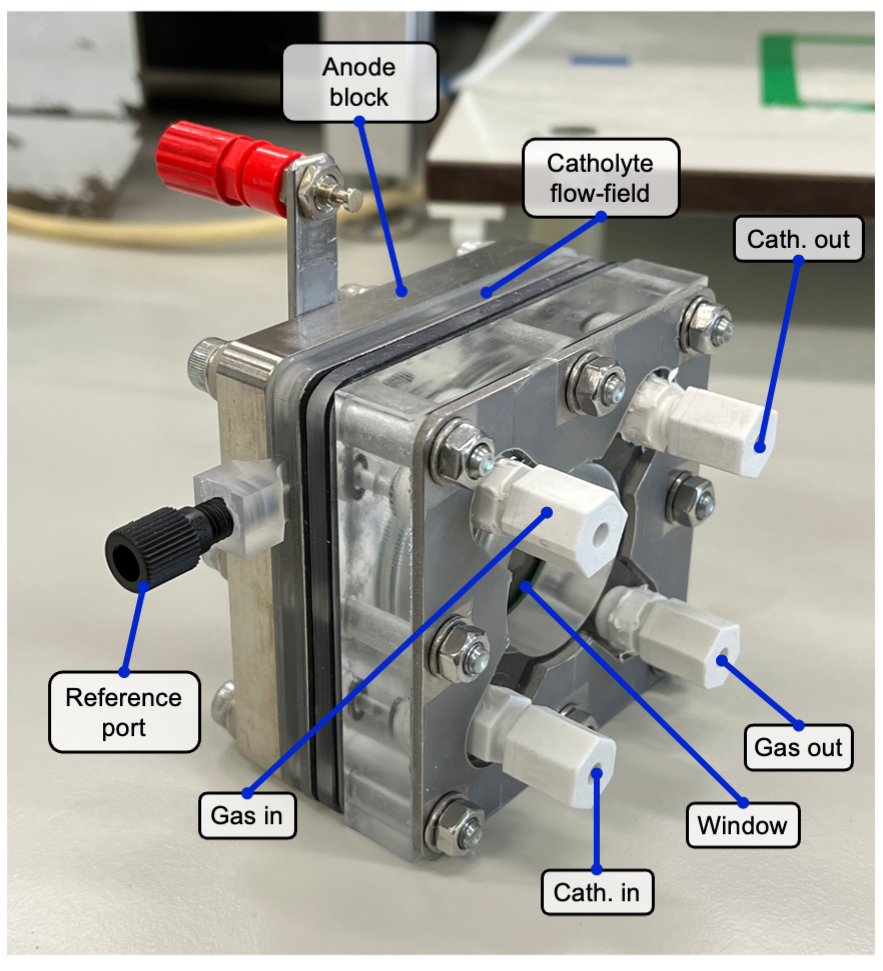


Supplementary Figure 5

State-of-the-art windowed electrolyzer used in the electrochemical characterization of the different cathodes. The assembly consists of a titanium anode block (with integrated flow-field), a catholyte flow-field (3D-printed), a gas chamber (3D-printed) and a window end-block (milled, PMMA) with a stainless-steel pressing plate.


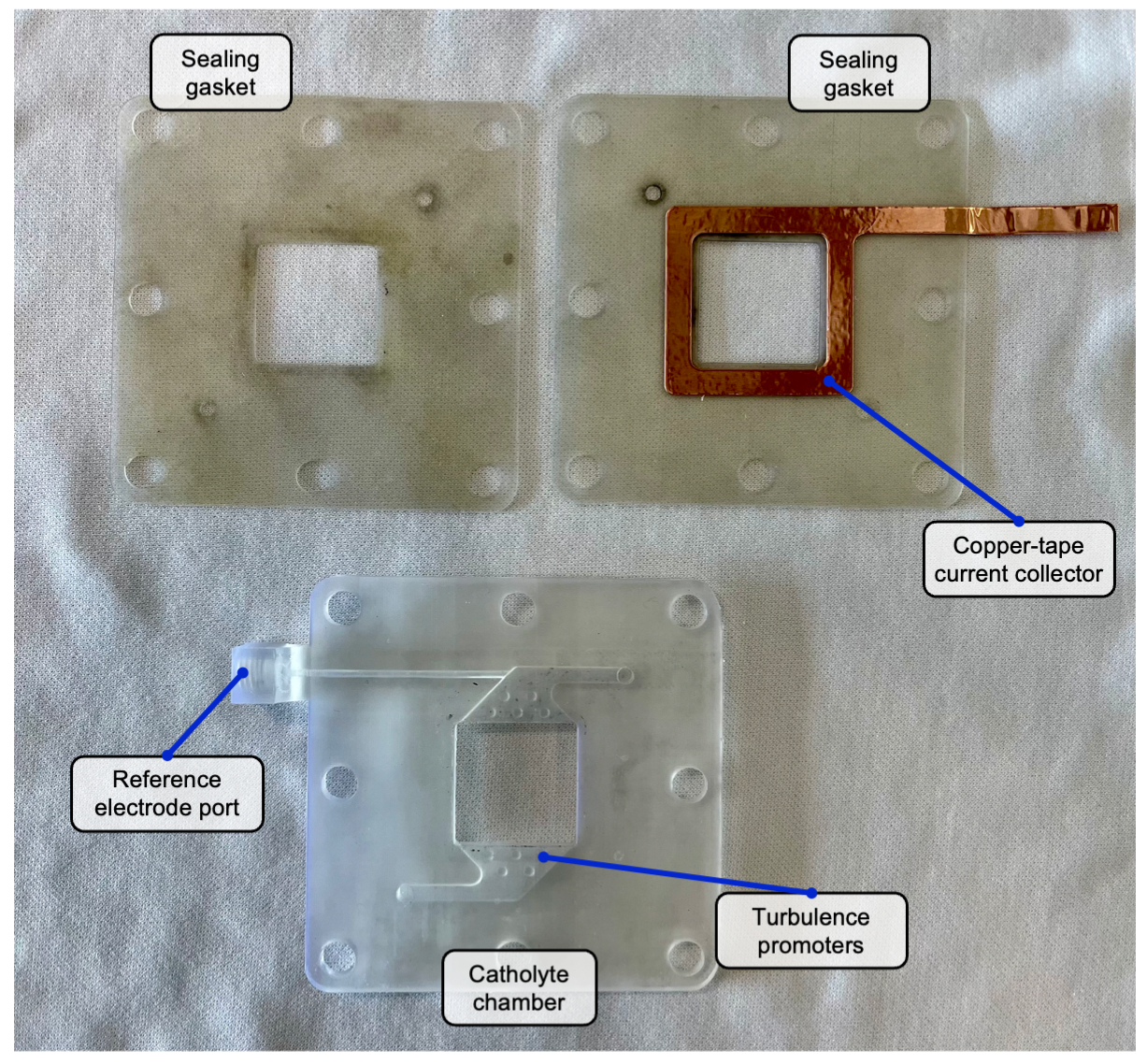


Supplementary Figure 6

Assembly around the cathode, consisting of a 3D-printed catholyte chamber with reference-electrode port and turbulence promotors, a sealing gasket (δ = 500 µm, silicone) with an edge-current collector (pressed to the silicone at 10 bar) and a sealing gasket (δ = 250 µm, silicone)


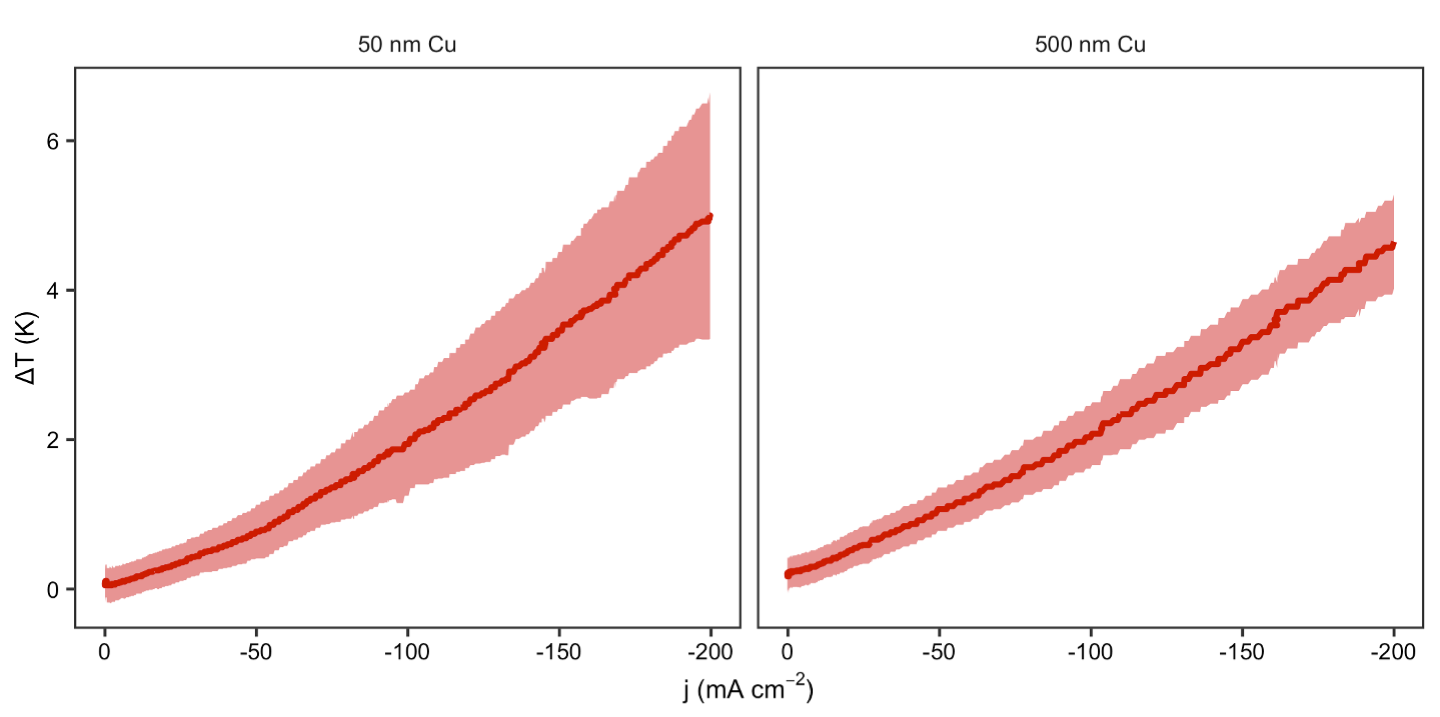


Supplementary Figure 7

Average (bold) and standard-deviation (shaded) temperature increase on 50 nm and 500 nm Cu ePTFE electrodes under increasing cathodic polarization.


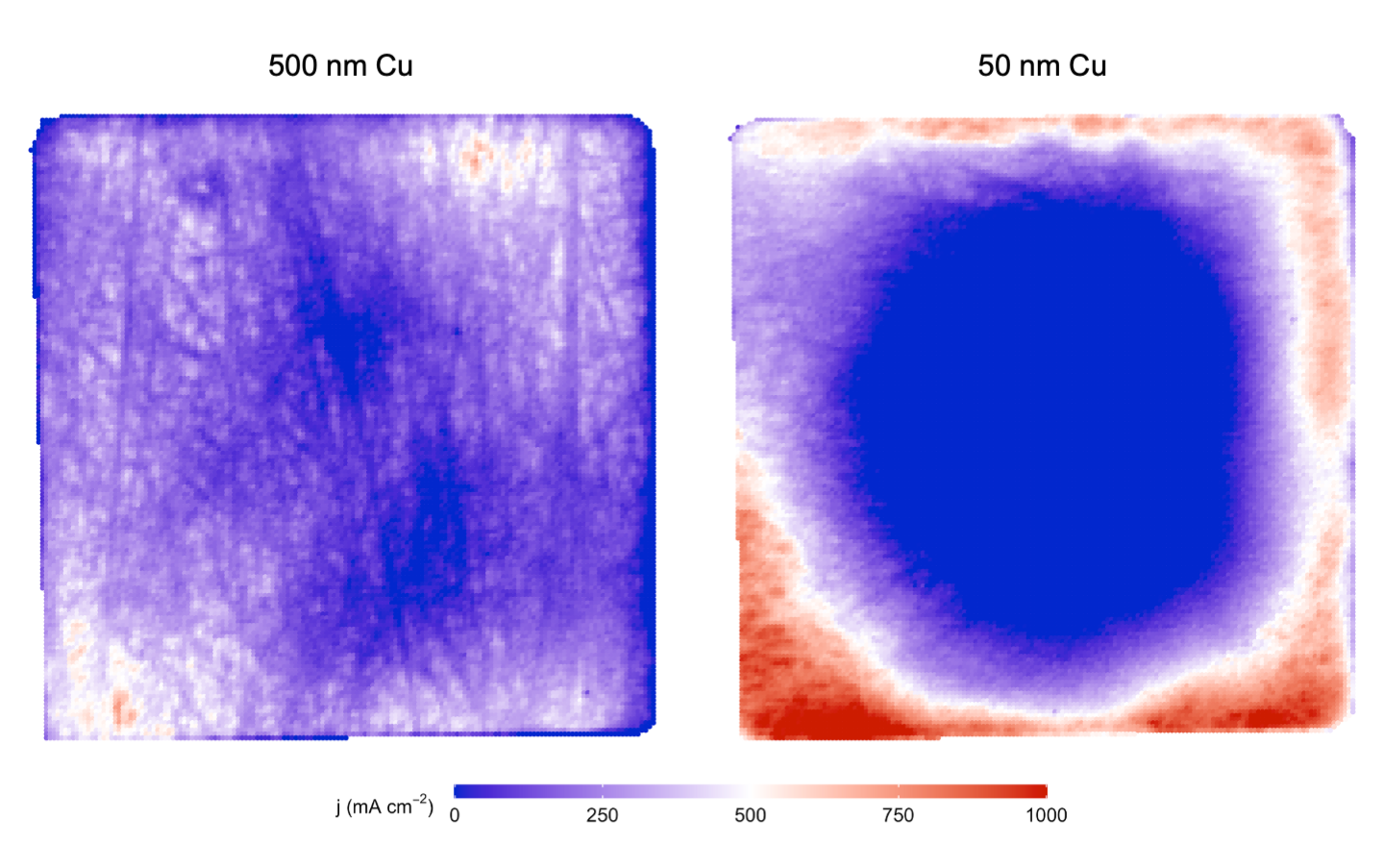


Supplementary Figure 8

Corrected local current densities based on the relative temperature increase per pixel for a 50 nm and a 500 nm Cu ePTFE electrode at –200 mA cm^-2^.


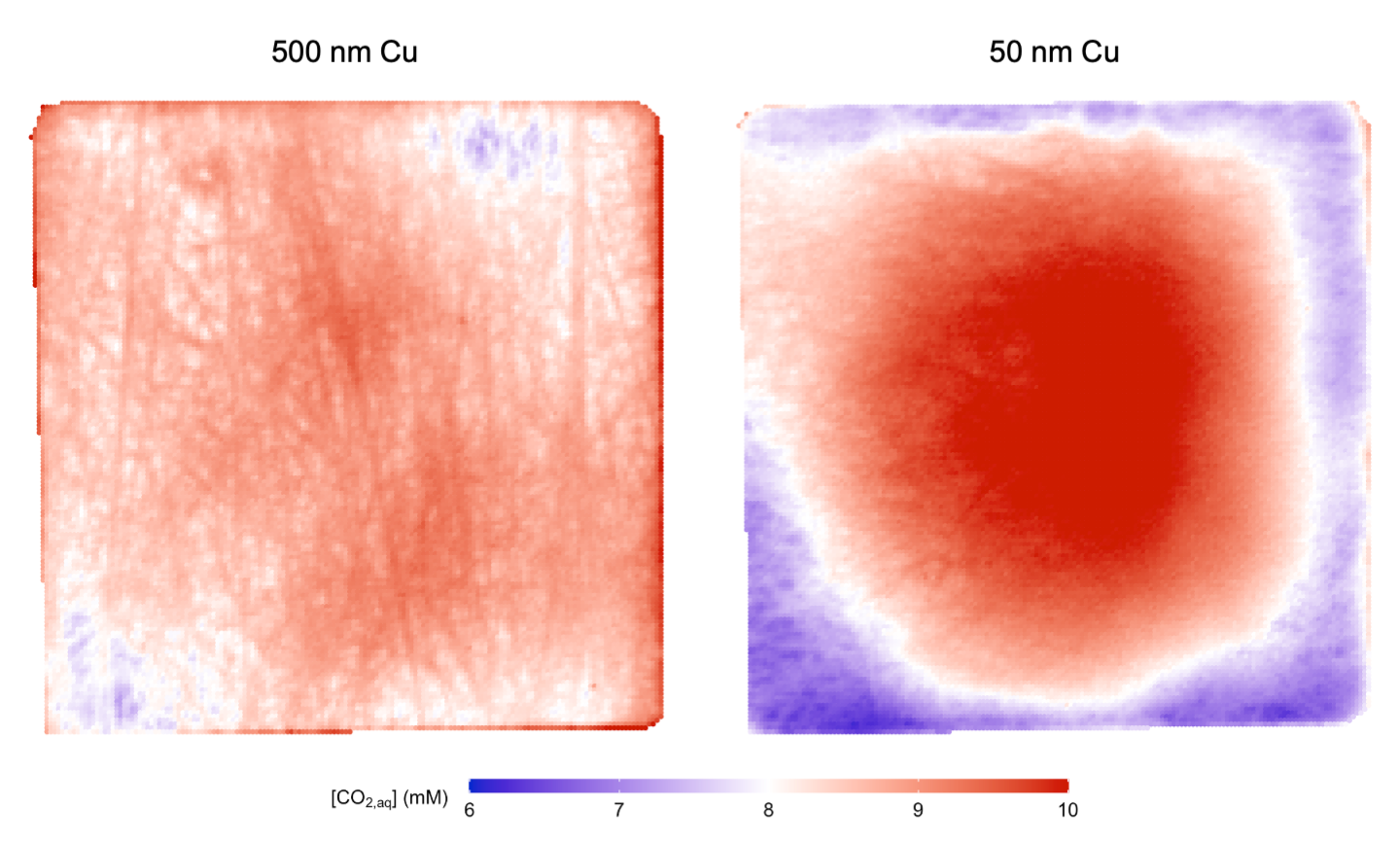


Supplementary Figure 9

Corrected local concentration of dissolved CO_2_ at the gas-liquid interface, based on the measured temperature per pixel for a 50 nm and a 500 nm Cu ePTFE electrode at an average *j* of –200 mA cm^-2^. Salting-out effects were calculated using interpolated ion-concentrations resulting from the model described in the Supplementary Text.


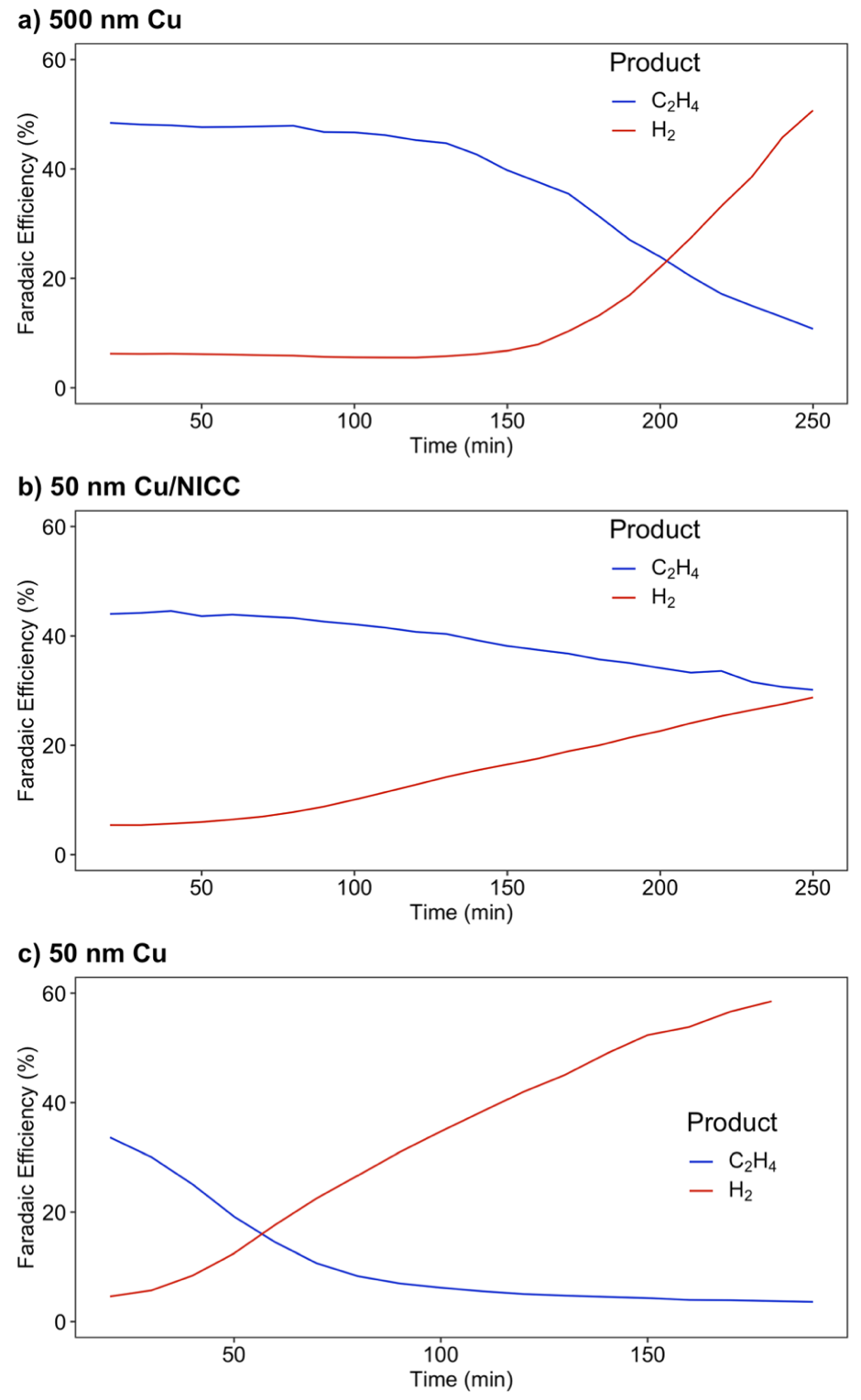


Supplementary Figure 10

Long-term faradaic efficiencies towards ethylene and hydrogen of a 500 nm Cu ePTFE electrode, a 50 nm Cu/NICC and a 50 nm Cu design, at constant potential (~ - 0.55 V vs. RHE)


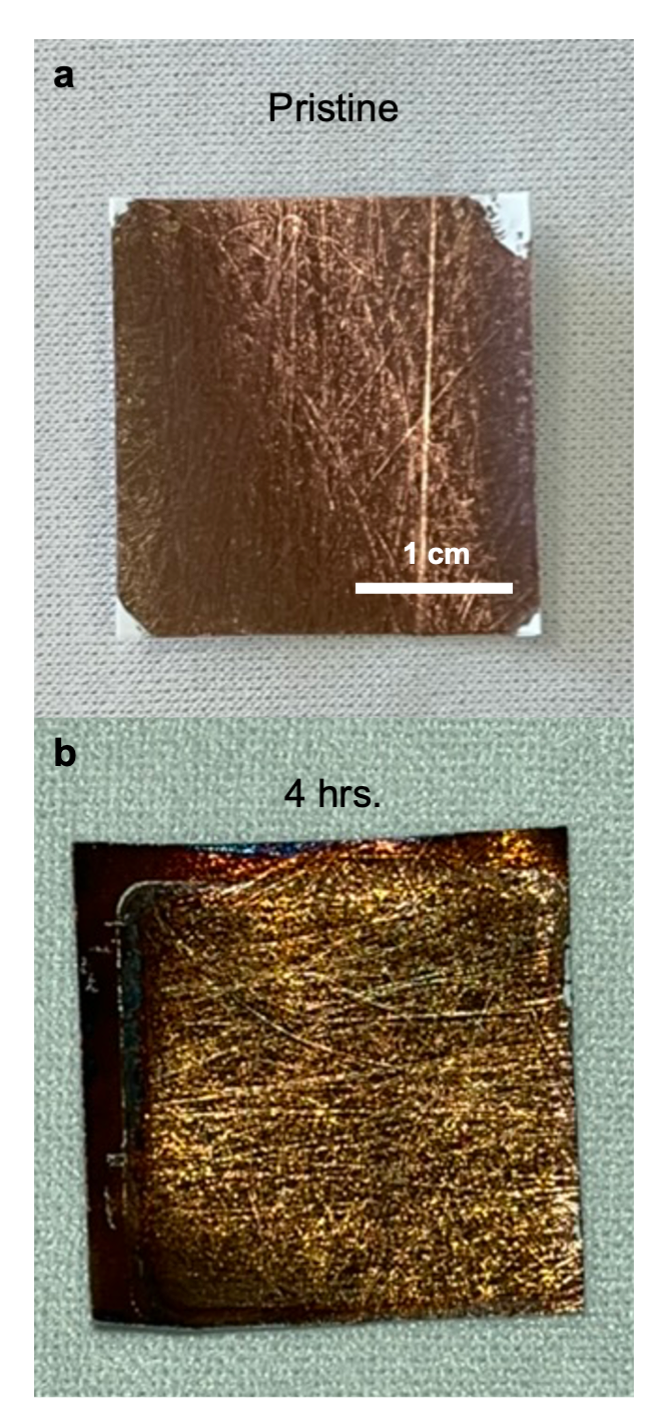


Supplementary Figure 11

(a) As-prepared 500 nm Cu ePTFE electrode, and (b) after 4 hrs. of electrolysis at ~-0.55 V vs. RHE


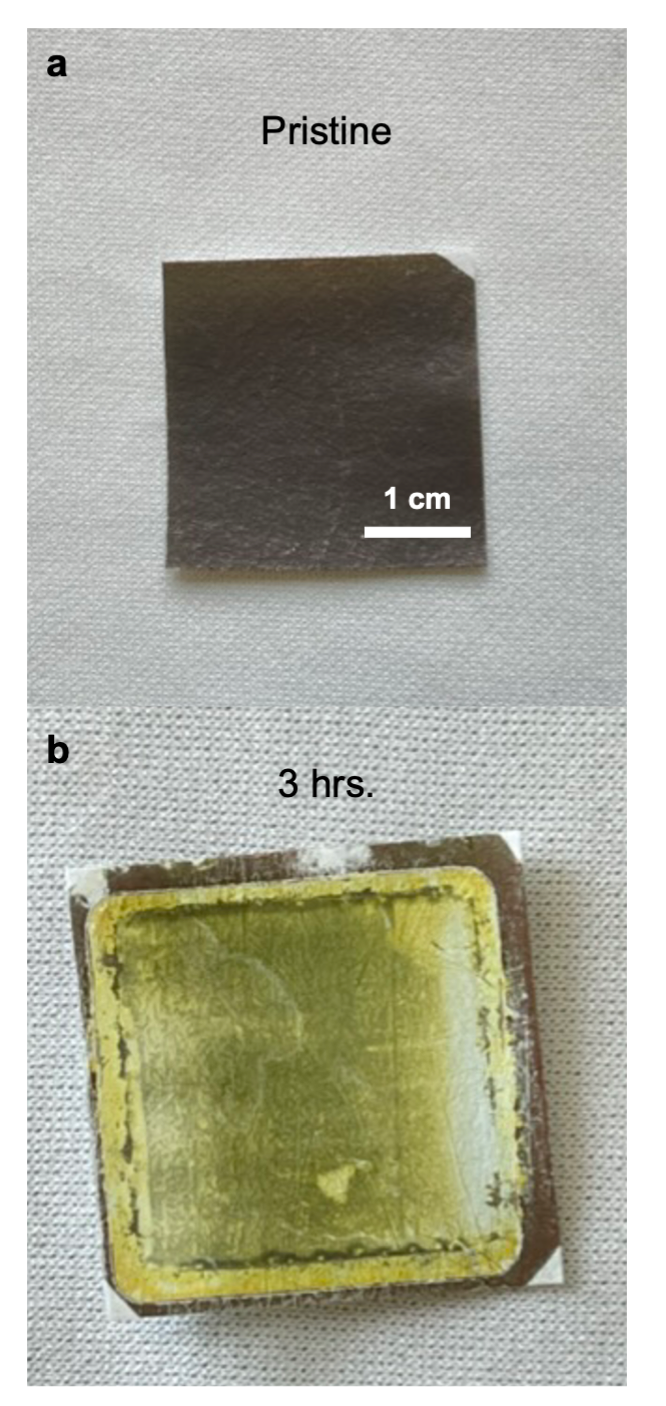


Supplementary Figure 12

(**a**) As-prepared 50 nm Cu ePTFE electrode, and (**b**) state of a 50 nm Cu sample after 3 hrs. of electrolysis at ~-0.55 V vs. RHE


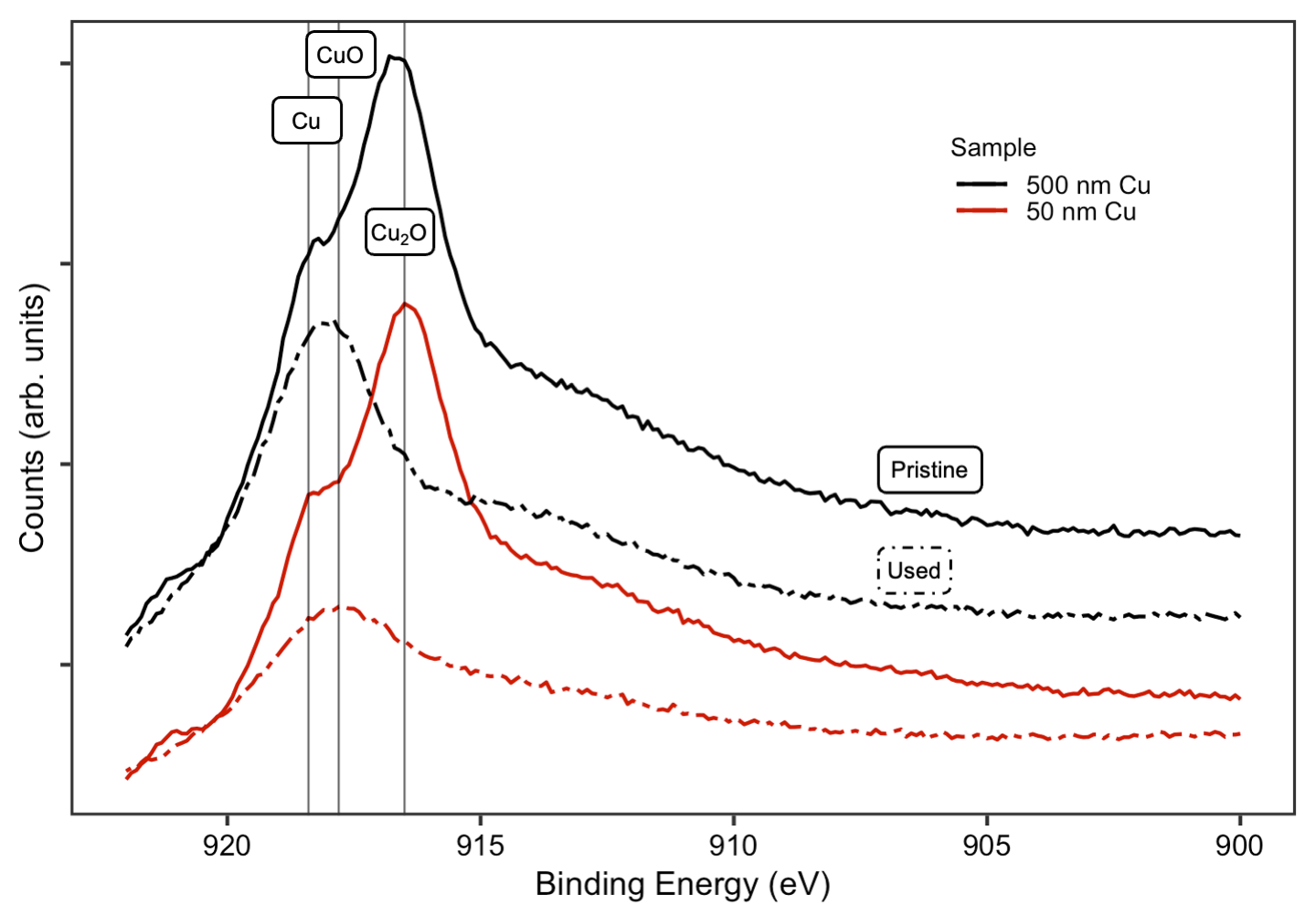


Supplementary Figure 13

Cu-LMM XPS scans of pristine and used samples of each thickness. The used 500 nm Cu scan shows a convolution of Cu and CuO peaks, whereas that convolution is much weaker for the 50 nm Cu used sample. The metallic copper shoulder is evident for both pristine samples.


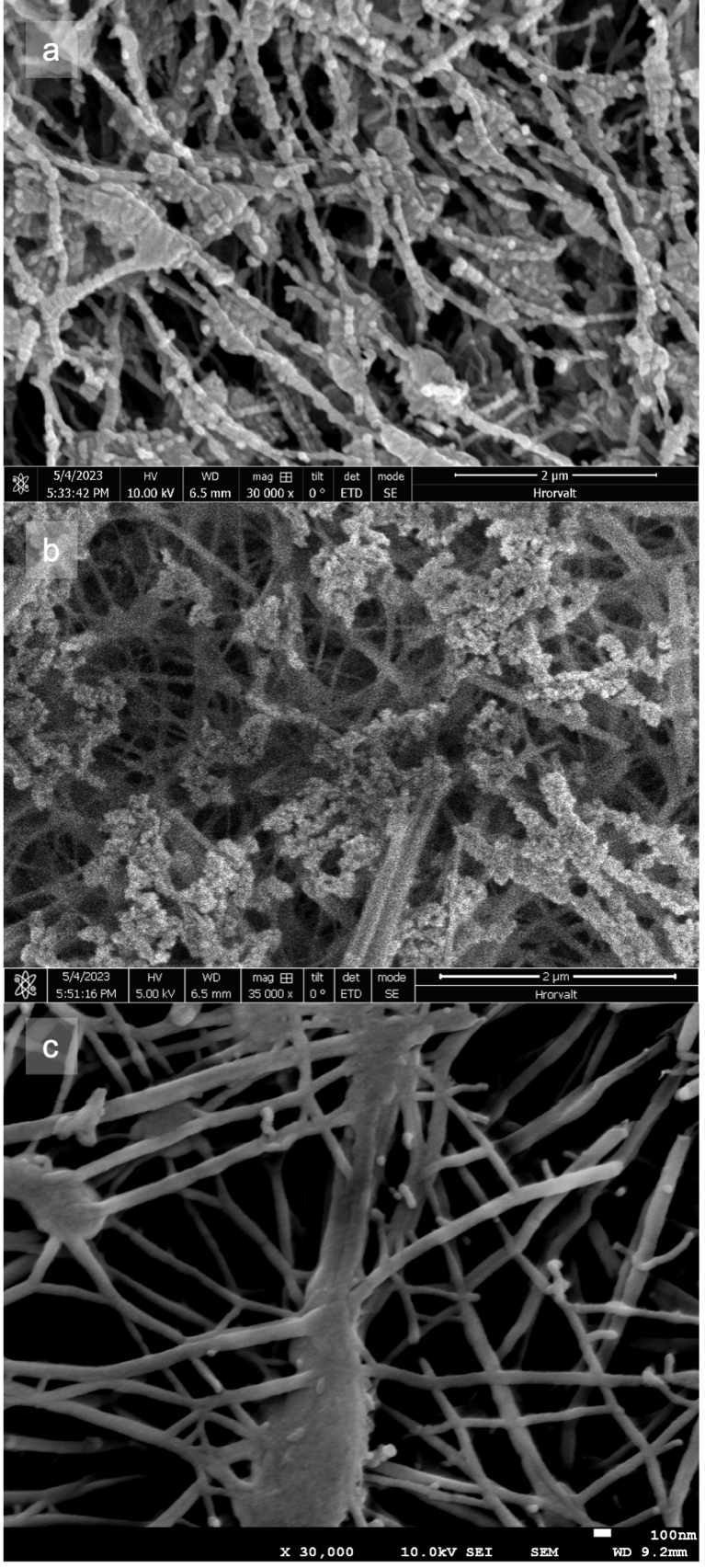


Supplementary Figure 14

(**a**) SEM imaging of as-prepared 50 nm Cu ePTFE electrode, (**b**) state of a 50 nm Cu electrode after prolonged (~ 2h) electrolysis at increasing current densities and (**c**) state of a bare spot of ePTFE after prolonged (~ 2h) electrolysis.


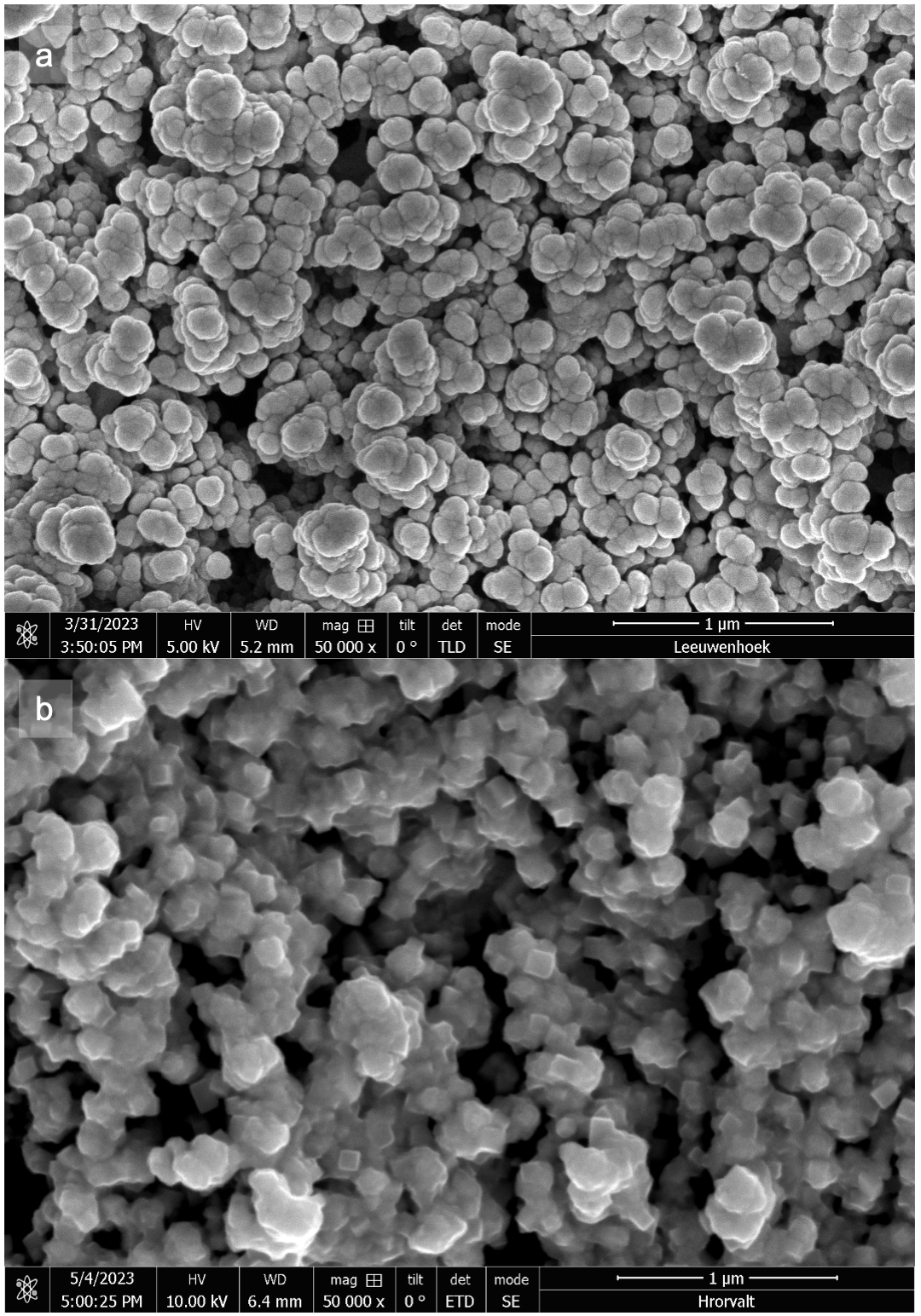


Supplementary Figure 15

(**a**) SEM imaging of as-prepared 200 nm Cu + Sigracet® 3BB electrode, and (**b**) after prolonged (~ 2h) electrolysis at increasing current densities from -50 to -200 mA cm^-2^.


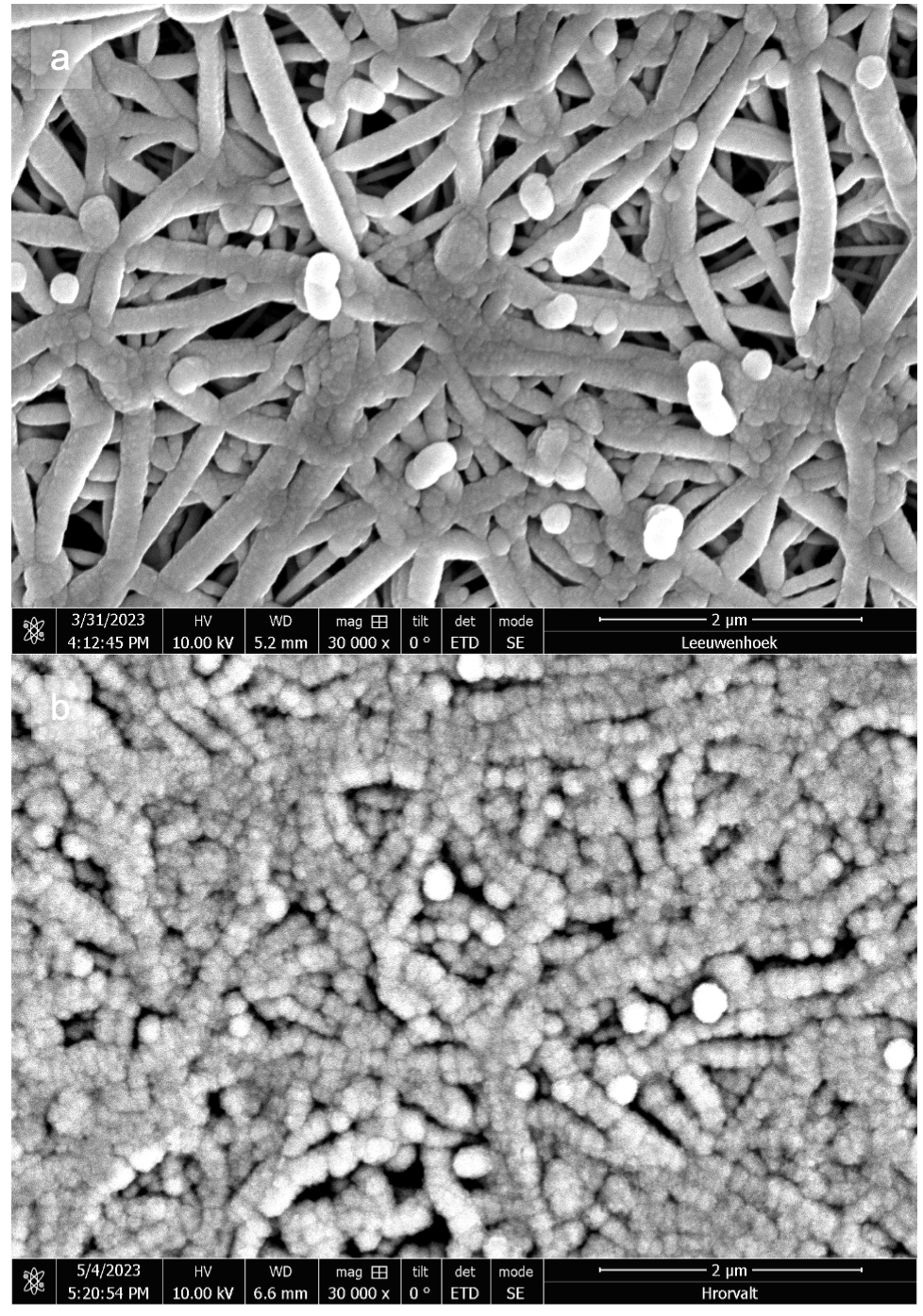


Supplementary Figure 16

(**a**) SEM imaging of as-prepared 500 nm Cu ePTFE electrode, and (**b**) after prolonged (~ 2h) electrolysis at increasing current densities from -50 to -200 mA cm^-2^.


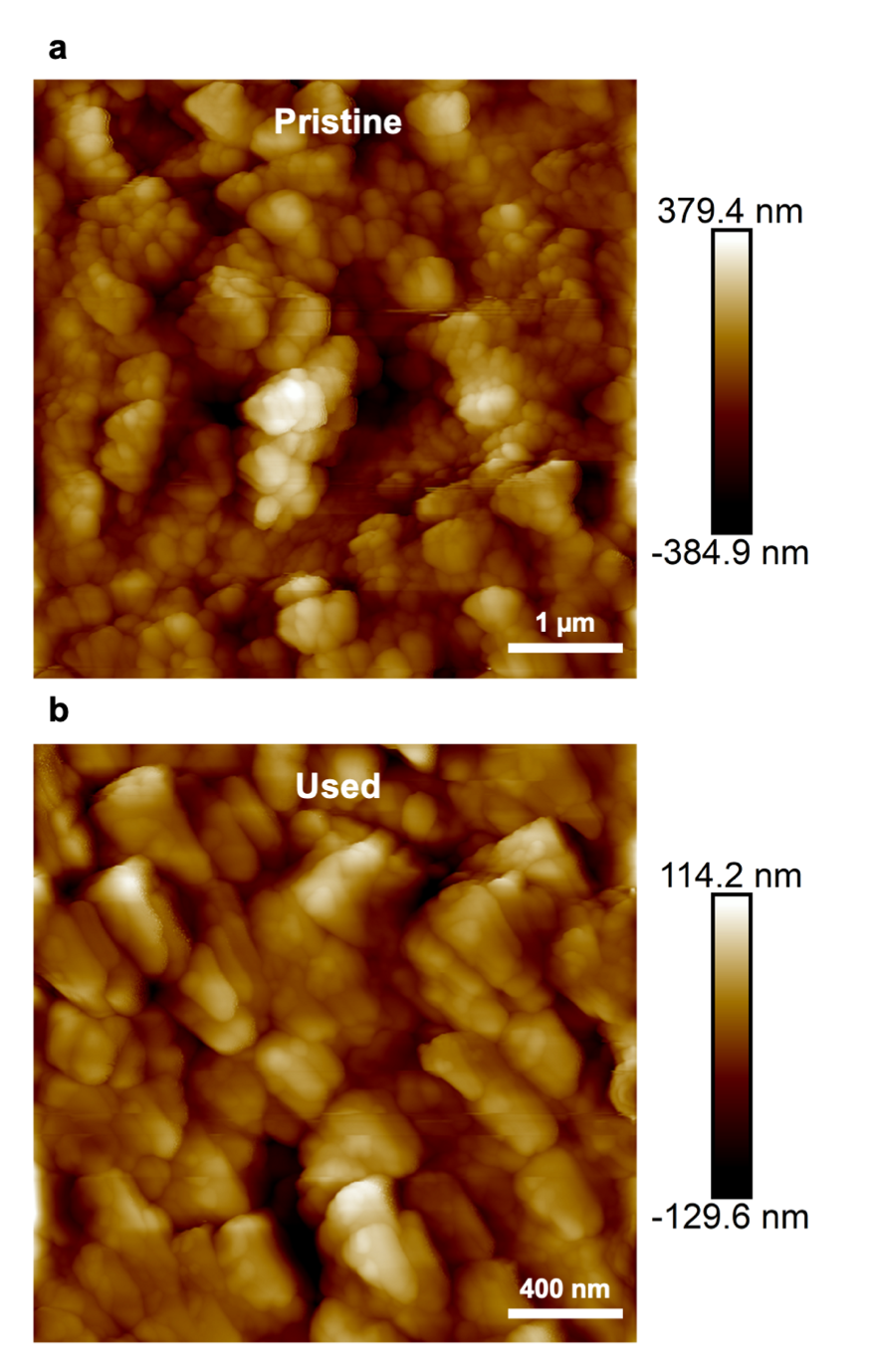


Supplementary Figure 17

AFM surface imaging of (**a**) as-prepared 200 nm Cu on Sigracet 38BB, and (**b**) state of the 200 nm Cu layer after prolonged (~ 2h) electrolysis at increasing current densities.


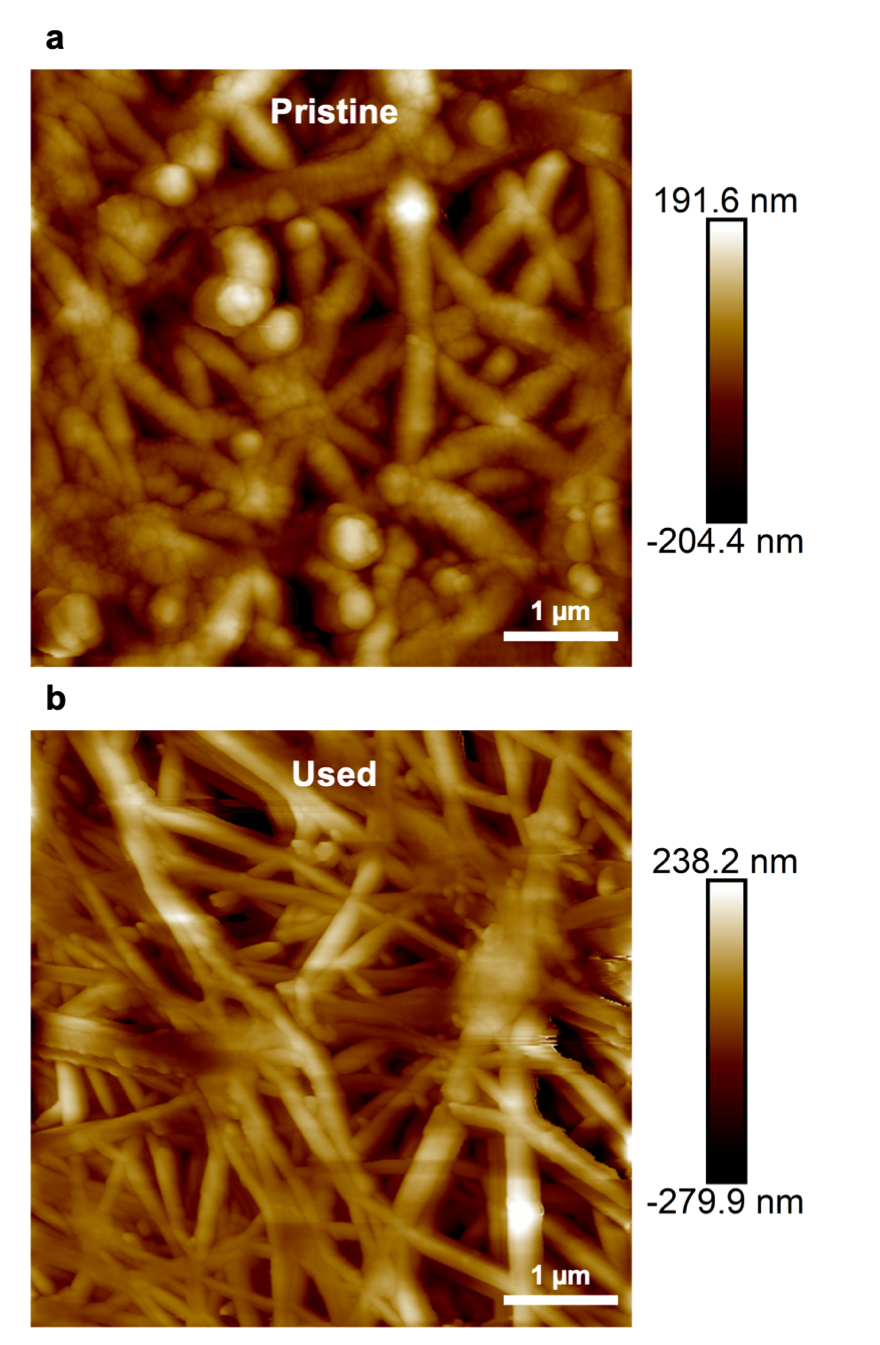


Supplementary Figure 18

AFM surface imaging of (**a**) as-prepared 50 nm Cu on ePTFE, and (**b**) state of the same layer after prolonged (~ 2h) electrolysis at increasing current densities.


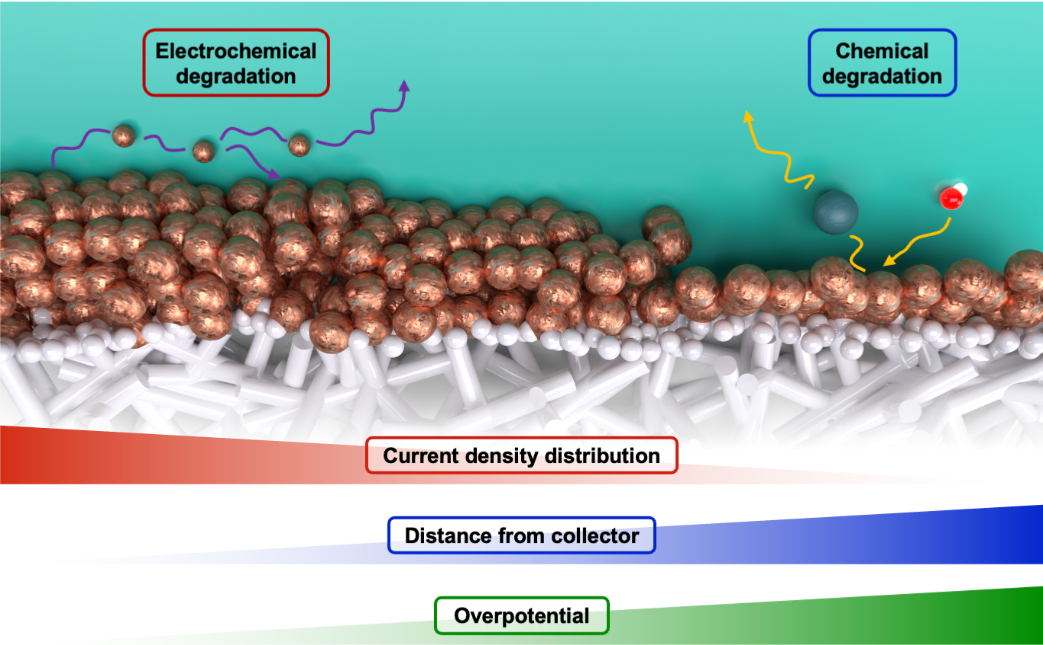


Supplementary Figure 19

Sketched deterioration mechanisms for sputtered copper catalyst layers on PTFE GDLs. Areas far from the current collector are progressively electrically isolated and experience chemical corrosion to soluble hydroxides. Areas close to the current collector experience cathodic corrosion resulting in resutructuring of the catalyst nanoparticles.


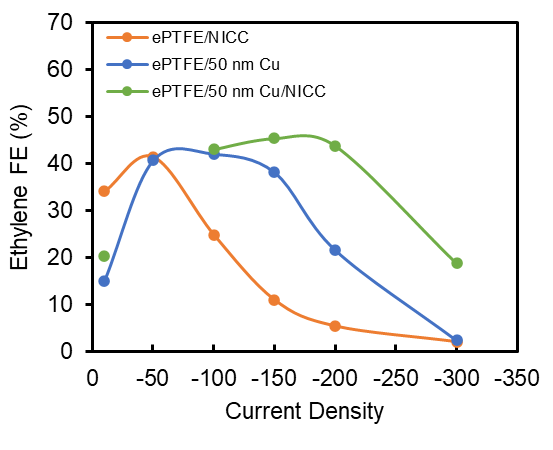


Supplementary Figure 20

Comparative ethylene Faradaic efficiency for different electrodes including: (1) ePTFE and a 1 µm busbar layer (NICC), (2) ePTFE + 50 nm Cu layer, (3) ePTFE + 50 nm Cu layer + a 1 µm busbar layer (NICC)


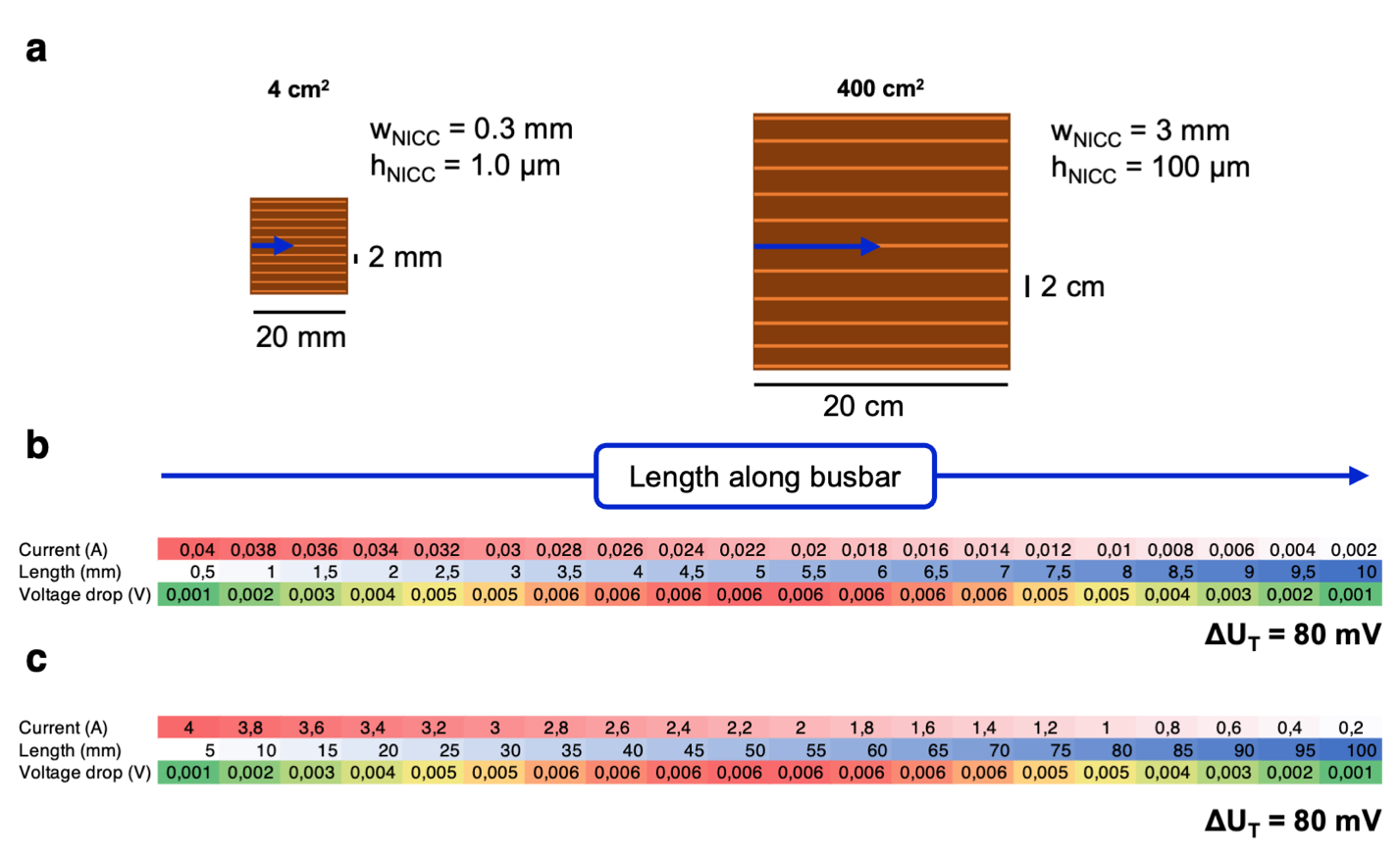


Supplementary Figure 21

(**a**) Sketches for NICC designs on 4 cm^2^ and 400 cm^2^ electrodes with detailed NICC dimensions. (**b**) The maximum voltage drop along the busbars for a current density of 200 mA cm^-2^ a 4 cm^2^ electrode as detailed in (a), and (**c**) a 400 cm^2^ electrode with a NICC design as in (a).


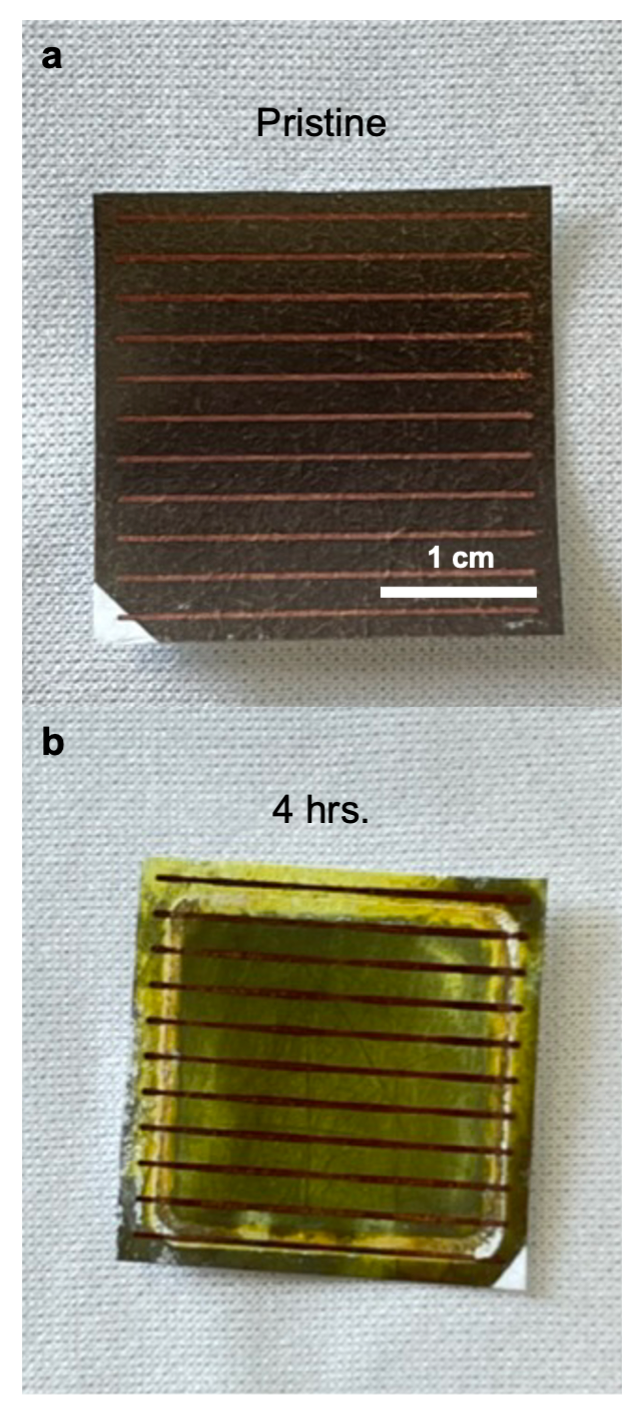


Supplementary Figure 22

(**a**) As-prepared 50 nm Cu/NICC ePTFE electrode, and (**b**) state of a NICC electrode after 4 hrs. of electrolysis at ~-0.55 V vs. RHE


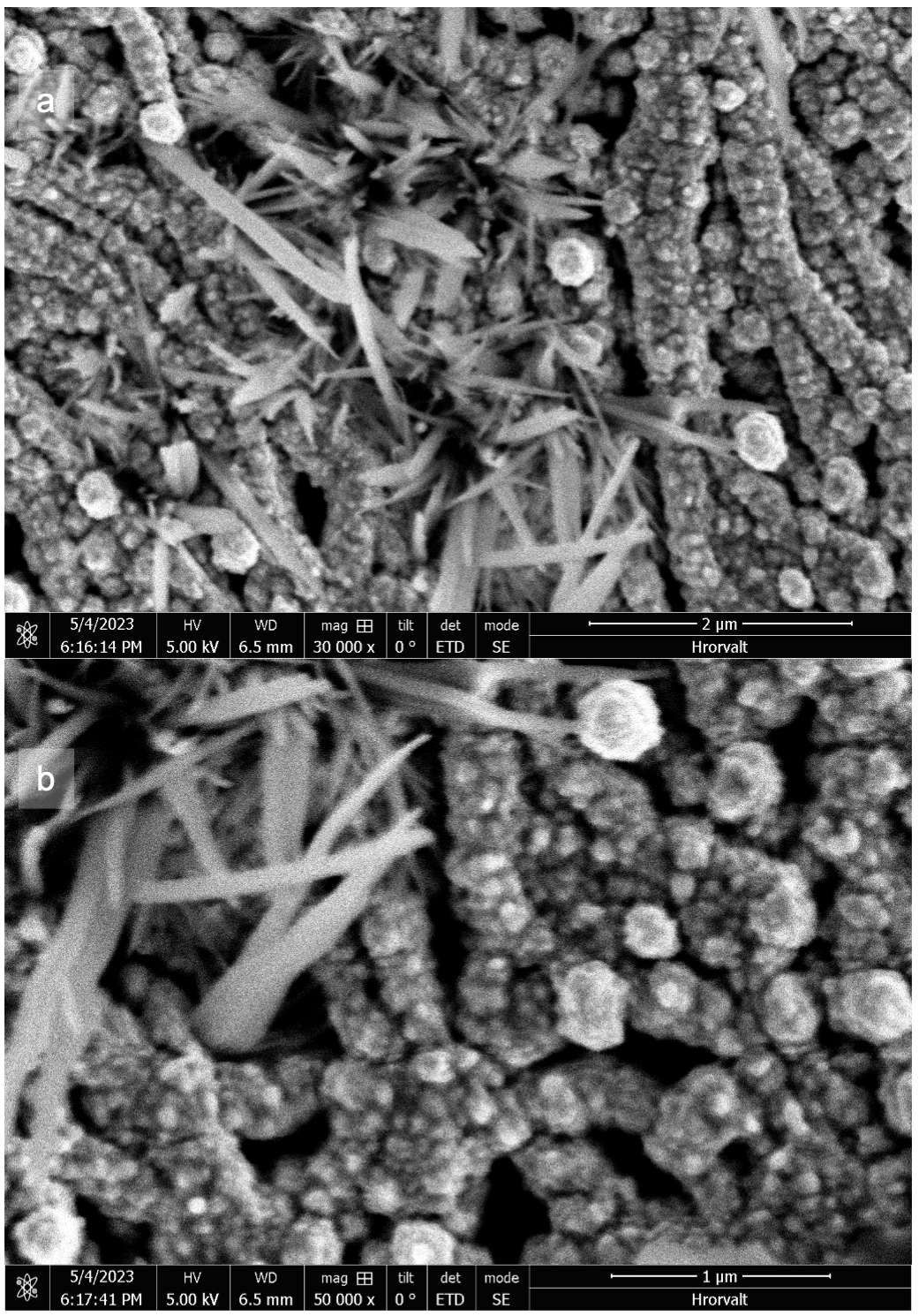


Supplementary Figure 23

(**a, b**) SEM imaging of a 50 nm Cu/NICC electrode after prolonged (~ 2h) electrolysis at increasing current densities. The crystalline structures are due to insufficient rinsing of potassium carbonates after running.


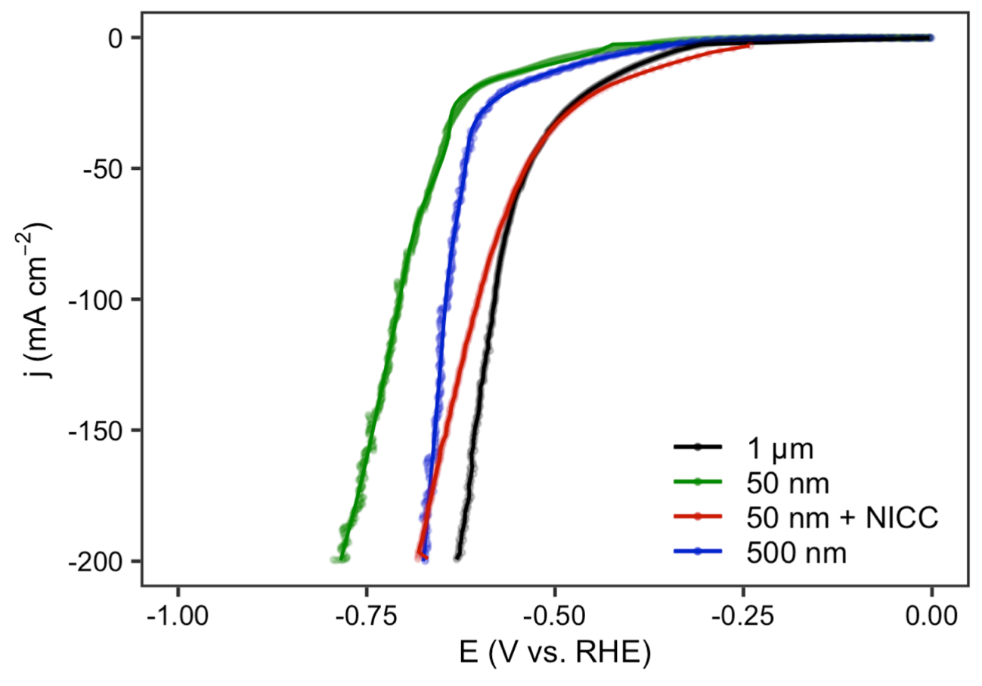


Supplementary Figure 24

Polarization curves at –5 mV s^-1^ of 1 µm, 500 nm, 50 nm and 50 nm Cu/NICC electrodes.


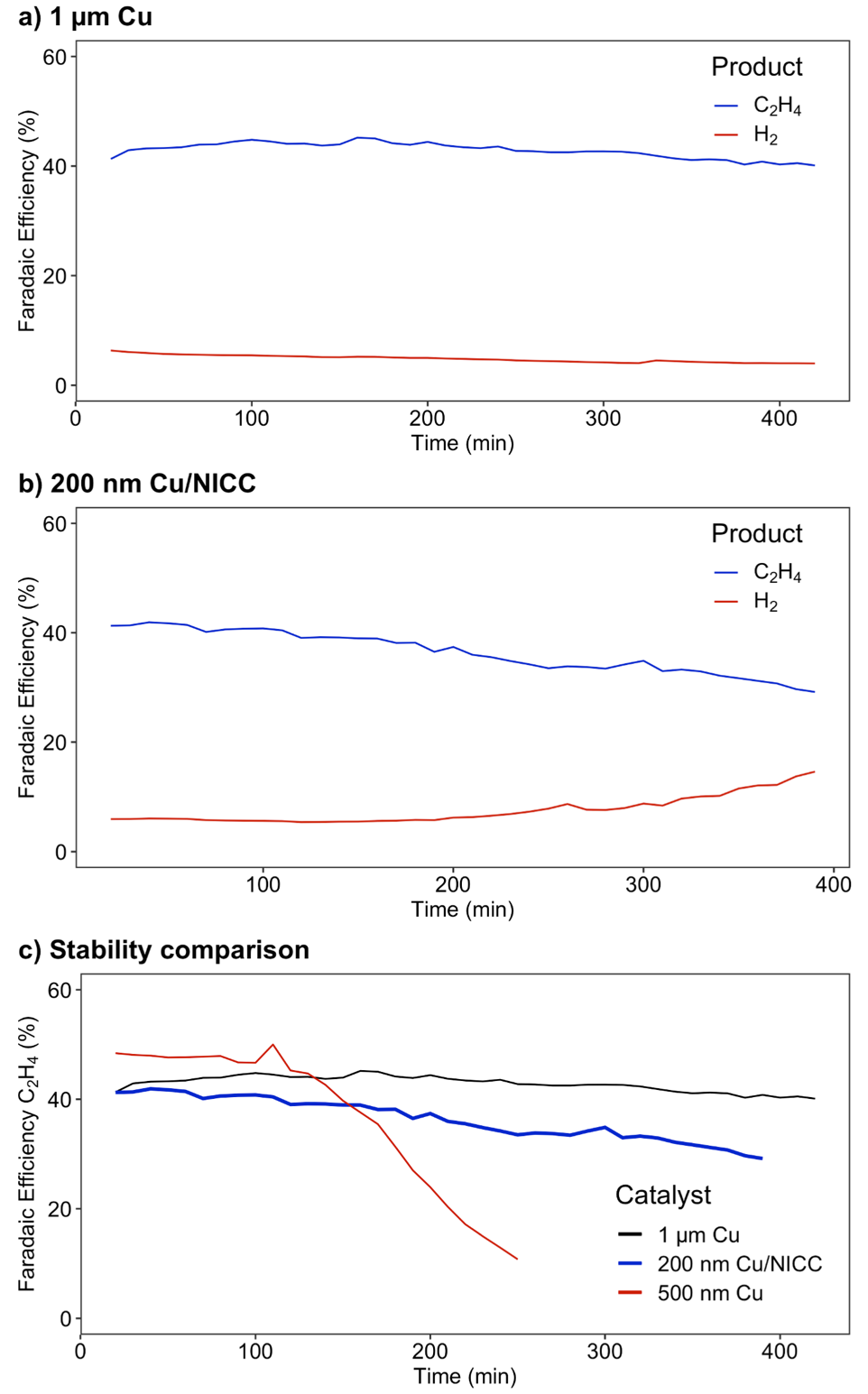


Supplementary Figure 25

Long-term faradaic efficiencies towards ethylene and hydrogen of (a) 1 µm Cu ePTFE electrode, (b) 200 nm Cu/NICC electrode, and (c) comparison of stability towards ethylene at constant potential (~ -0.6 V vs. RHE).

Supplementary Table 3

2-electrode probe resistances measured for a 50 nm and a 1 µm Cu layers.

| **Cu layer thickness** | **R_path,1cm_ [mΩ cm]** |
| --- | --- |
| 50 nm | 1.47 |
| 1 µm | 0.17 |

Supplementary Table 4

Selectivities for a 1 µm, 500 nm and 50 nm catalyst layers sputtered on ePTFE GDEs, and of a 200 nm Cu layer on a carbonous electrode (Sigracet® 38BB). Data taken at increased current densities. FE’s of liquid products are corrected and approximated to resting electrolyte volume at time of acquisition.

|  |  | **Faradaic Efficiency [%]** | | | | | | |  | | |
| --- | --- | --- | --- | --- | --- | --- | --- | --- | --- | --- | --- |
|  | **j  [mA cm^-2^]** | Methane [CH_4_] | Formate [HCOO^-^] | Ethylene [C_2_H_4_] | Ethanol [C_2_H_5_OH] | Acetate [CH_3_COO^-^] | Propanol [CH_3_H_7_OH] | Acetaldehyde [C_2_H_4_O] | **C_1_** | **C_2+_** | **H_2_** |
| **1 µm Cu** | **– 10** | 0.00 | 22.1 | 9.65 | 0.48 | 2.62 | 0.11 | 0.00 | **22.1** | **12.9** | **8.73** |
|  | **– 50** | 0.00 | 10.8 | 33.5 | 13.0 | 0.57 | 6.83 | 0.00 | **10.8** | **53.9** | **8.42** |
|  | **– 100** | 0.00 | 7.35 | 43.5 | 14.2 | 0.95 | 6.07 | 0.00 | **7.35** | **64.7** | **5.83** |
|  | **– 150** | 0.00 | 7.92 | 46.5 | 25.3 | 1.38 | 9.22 | 7.85 | **7.92** | **90.3** | **4.45** |
|  | **– 200** | 0.00 | 6.26 | 48.3 | 31.0 | 2.61 | 8.62 | 7.07 | **6.26** | **97.6** | **3.87** |
|  | **– 300** | 0.00 | 5.13 | 47.9 | 32.3 | 3.27 | 6.80 | 8.51 | **5.29** | **98.8** | **4.87** |
| **500 nm Cu** | **– 10** | 1.11 | 16.0 | 17.8 | 15.0 | 7.40 | 0.13 | 0.14 | **17.1** | **40.5** | **16.1** |
|  | **– 50** | 0.20 | 5.79 | 40.4 | 14.8 | 1.30 | 9.03 | 2.91 | **5.99** | **68.5** | **9.92** |
|  | **– 100** | 0.30 | 4.70 | 45.5 | 23.5 | 2.19 | 6.79 | 3.58 | **5.00** | **81.5** | **6.50** |
|  | **– 150** | 0.30 | 3.98 | 45.2 | 30.9 | 2.35 | 8.46 | 3.99 | **4.27** | **90.9** | **5.70** |
|  | **– 200** | 0.33 | 3.59 | 45.8 | 33.5 | 2.97 | 6.97 | 3.63 | **3.91** | **92.8** | **6.05** |
|  | **– 300** | 4.85 | 3.00 | 29.8 | 43.5 | 6.79 | 5.42 | 4.32 | **7.84** | **89.8** | **18.5** |
| **50 nm Cu** | **– 10** | 0.42 | 16.3 | 15.1 | 0.71 | 8.30 | 0.11 | 0.12 | **16.7** | **24.3** | **21.2** |
|  | **– 50** | 4.64 | 5.08 | 40.7 | 14.9 | 2.57 | 5.59 | 6.39 | **9.72** | **70.1** | **6.83** |
|  | **– 100** | 6.59 | 4.66 | 42.1 | 19.5 | 5.76 | 4.11 | 6.36 | **11.3** | **77.8** | **4.73** |
|  | **– 150** | 13.3 | 3.99 | 38.2 | 24.4 | 7.90 | 4.49 | 6.47 | **17.3** | **81.5** | **6.42** |
|  | **– 200** | 31.6 | 4.61 | 21.7 | 21.6 | 9.26 | 2.72 | 4.76 | **36.2** | **60.0** | **17.2** |
|  | **– 300** | 29.4 | 4.90 | 2.46 | 13.4 | 5.12 | 2.83 | 3.63 | **34.3** | **27.5** | **30.8** |
| **200 nm Cu/C** | **– 10** | 0.15 | 30.8 | 2.59 | 11.8 | 6.41 | 0.11 | 0.12 | **31.0** | **19.8** | **28.6** |
|  | **– 50** | 2.48 | 9.05 | 20.6 | 5.90 | 0.87 | 3.25 | 0.12 | **11.5** | **31.3** | **13.5** |
|  | **– 100** | 4.15 | 8.79 | 32.3 | 17.2 | 2.32 | 4.86 | 3.59 | **12.9** | **61.2** | **9.13** |
|  | **– 150** | 4.15 | 5.81 | 36.2 | 19.2 | 2.90 | 5.19 | 2.97 | **12.7** | **67.3** | **7.70** |
|  | **– 200** | 5.13 | 5.99 | 37.2 | 25.6 | 4.25 | 4.76 | 4.06 | **11.1** | **76.5** | **7.98** |
|  | **– 300** | 6.45 | 4.00 | 24.4 | 25.4 | 5.80 | 3.74 | 2.83 | **10.5** | **62.3** | **9.36** |

Supplementary Table 5

Selectivity for a uniform 50 nm Cu catalyst layers sputtered on an ePTFE GDEs, of a 1 µm NICC pattern sputtered on a bare GDE and of a 50 nm Cu/NICC electrode. Data taken at increased current densities.

|  |  | **Faradaic Efficiency [%]** | | | | |  |  |  |
| --- | --- | --- | --- | --- | --- | --- | --- | --- | --- |
|  | **j  [mA cm^-2^]** | | Methane [CH_4_] | Ethylene [C_2_H_4_] | Hydrogen [H_2_] | Carbon Monoxide [CO] | | Propane [C_3_H_8_] | **Gas products** |
| **Bare/NICC** | **– 10** | | 0.00 | 34.1 | 4.15 | 20.3 | | 0.50 | **59.1** |
|  | **– 50** | | 0.72 | 41.5 | 3.76 | 4.18 | | 0.57 | **50.7** |
|  | **– 100** | | 6.23 | 24.9 | 25.2 | 0.86 | | 0.12 | **57.4** |
|  | **– 150** | | 9.10 | 11.0 | 54.5 | 0.33 | | 0.03 | **75.0** |
|  | **– 200** | | 5.09 | 5.42 | 69.6 | 0.26 | | 0.02 | **80.4** |
|  | **– 300** | | 1.19 | 2.05 | 71.9 | 0.24 | | 0.01 | **75.4** |
| **50 nm Cu** | **– 10** | | 0.42 | 15.1 | 21.2 | 35.7 | | 0.37 | **72.8** |
|  | **– 50** | | 4.64 | 40.7 | 6.83 | 8.93 | | 0.60 | **61.7** |
|  | **– 100** | | 6.59 | 42.1 | 4.73 | 5.06 | | 0.58 | **59.0** |
|  | **– 150** | | 13.3 | 38.2 | 6.42 | 1.65 | | 0.38 | **60.0** |
|  | **– 200** | | 31.6 | 21.7 | 17.2 | 0.62 | | 0.17 | **71.3** |
|  | **– 300** | | 29.4 | 2.46 | 30.8 | 0.09 | | 0.03 | **62.8** |
| **50 nm Cu/NICC** | **– 10** | | 0.39 | 20.4 | 8.98 | 25.7 | | 0.36 | **55.8** |
|  | **– 100** | | 3.04 | 43.1 | 5.48 | 9.32 | | 0.42 | **61.3** |
|  | **– 150** | | 3.26 | 45.4 | 4.47 | 6.45 | | 0.77 | **60.4** |
|  | **– 200** | | 5.63 | 43.9 | 4.57 | 4.29 | | 0.54 | **58.9** |
|  | **– 300** | | 27.5 | 18.8 | 20.54 | 6.59 | | 0.10 | **73.6** |

Supplementary Table 6

Faradaic efficiencies towards ethylene, hydrogen, and carbon monoxide of the architectures compared in Figure 4. All data is taken at around –0.55 V vs. RHE using the setup described previously.

|  | **500 nm Cu** | | | **50 nm Cu/NICC** | | | **50 nm Cu** | | |
| --- | --- | --- | --- | --- | --- | --- | --- | --- | --- |
| **t [min]** | FE C_2_H_4_ [%] | FE H_2_ [%] | FE CO [%] | FE C_2_H_4_ [%] | FE H_2_ [%] | FE CO [%] | FE C_2_H_4_ [%] | FE H_2_ [%] | FE CO [%] |
| **10** | 48,42 | 6,26 | 4,36 | 44,03 | 5,42 | 5,92 | 33,62 | 4,62 | 12,90 |
| **20** | 48,11 | 6,21 | 4,17 | 44,21 | 5,41 | 5,99 | 30,06 | 5,72 | 13,48 |
| **30** | 47,97 | 6,25 | 4,38 | 44,57 | 5,67 | 5,85 | 25,08 | 8,43 | 14,37 |
| **40** | 47,64 | 6,17 | 4,54 | 43,62 | 5,97 | 5,37 | 19,18 | 12,47 | 14,99 |
| **50** | 47,67 | 6,09 | 4,65 | 43,90 | 6,44 | 4,97 | 14,49 | 17,70 | 15,43 |
| **60** | 47,77 | 5,98 | 4,75 | 43,58 | 6,98 | 4,58 | 10,68 | 22,53 | 15,46 |
| **70** | 47,89 | 5,90 | 4,86 | 43,30 | 7,78 | 4,31 | 8,31 | 26,72 | 15,14 |
| **80** | 46,73 | 5,68 | 4,85 | 42,62 | 8,79 | 4,12 | 7,00 | 30,94 | 15,11 |
| **90** | 46,67 | 5,60 | 4,83 | 42,12 | 10,06 | 4,10 | 6,22 | 34,73 | 14,76 |
| **100** | 46,17 | 5,57 | 4,86 | 41,52 | 11,42 | 4,03 | 5,56 | 38,37 | 14,18 |
| **110** | 45,26 | 5,56 | 4,90 | 40,75 | 12,79 | 3,94 | 5,06 | 41,98 | 13,73 |
| **120** | 44,70 | 5,80 | 4,97 | 40,38 | 14,20 | 3,90 | 4,77 | 45,07 | 13,04 |
| **130** | 42,63 | 6,17 | 5,01 | 39,20 | 15,43 | 3,81 | 4,54 | 48,92 | 12,68 |
| **140** | 39,75 | 6,78 | 4,87 | 38,16 | 16,51 | 4,13 | 4,32 | 52,33 | 12,12 |
| **150** | 37,60 | 7,90 | 4,91 | 37,44 | 17,58 | 4,04 | 3,98 | 53,81 | 11,40 |
| **160** | 35,46 | 10,34 | 5,17 | 36,78 | 18,94 | 4,03 | 3,92 | 56,62 | 11,04 |
| **170** | 31,33 | 13,21 | 5,27 | 35,71 | 20,00 | 3,94 | 3,77 | 58,51 | 10,48 |
| **180** | 27,03 | 16,93 | 5,23 | 35,05 | 21,41 | 3,93 | 3,62 | 60,50 | 10,06 |
| **190** | 23,94 | 22,04 | 5,04 | 34,14 | 22,62 | 3,91 | - | - | - |
| **200** | 20,36 | 27,39 | 4,74 | 33,29 | 24,06 | 3,89 | - | - | - |
| **210** | 17,16 | 33,20 | 4,37 | 33,59 | 25,33 | 4,05 | - | - | - |
| **220** | 14,98 | 38,57 | 3,94 | 31,59 | 26,46 | 3,90 | - | - | - |
| **230** | 12,90 | 45,76 | 3,61 | 30,68 | 27,53 | 3,89 | - | - | - |
| **240** | 10,77 | 50,70 | 3,18 | 30,15 | 28,75 | 3,89 | - | - | - |

Supplementary References

1. Guyer, J. E., Wheeler, D. & Warren, J. A. FiPy: Partial Differential Equations with Python. *Comput. Sci. Eng.* **11**, 6–15 (2009).

2. Tiwari, P., Tsekouras, G., Swiegers, G. F. & Wallace, G. G. Gortex-Based Gas Diffusion Electrodes with Unprecedented Resistance to Flooding and Leaking. *ACS Appl. Mater. Interfaces* **10**, 28176–28186 (2018).

3. Samu, A. A., Szenti, I., Kukovecz, Á., Endrődi, B. & Janáky, C. Systematic screening of gas diffusion layers for high performance CO2 electrolysis. *Commun. Chem.* **6**, 41 (2023).

4. Weisenberger, S. & Schumpe, A. Estimation of gas solubilities in salt solutions at temperatures from 273 K to 363 K. *AIChE J.* **42**, 298–300 (1996).

5. Dinh, C. T. *et al.* CO2 electroreduction to ethylene via hydroxide-mediated copper catalysis at an abrupt interface. *Science* **360**, 783–787 (2018).

6. García de Arquer, F. P. *et al.* CO2 electrolysis to multicarbon products at activities greater than 1 A cm−2. *Science* **367**, 661–666 (2020).

7. Wiesenburg, D. A. & Guinasso, N. L. Equilibrium solubilities of methane, carbon monoxide, and hydrogen in water and sea water. *J. Chem. Eng. Data* **24**, 356–360 (1979).

8. Blake, J. W., Padding, J. T. & Haverkort, J. W. Analytical modelling of CO 2 reduction in gas-diffusion electrode catalyst layers. *Electrochimica Acta* **393**, 138987 (2021).

9. Iglesias van Montfort, H.-P. & Burdyny, T. Mapping Spatial and Temporal Electrochemical Activity of Water and CO2 Electrolysis on Gas-Diffusion Electrodes Using Infrared Thermography. *ACS Energy Lett.* **7**, 2410–2419 (2022).

10. Overview of materials for Polytetrafluoroethylene (PTFE), Extruded. https://www.matweb.com/search/datasheet_print.aspx?matguid=4e0b2e88eeba4aaeb18e8820f1444cdb.

11. Deen, W. M. Analysis of transport phenomena. (1998).
